# Melittin-loaded Iron Oxide Nanoparticles Prevent Intracranial Arterial Dolichoectasia Development through Inhibition of Macrophage-mediated Inflammation

**DOI:** 10.7150/ijbs.60588

**Published:** 2021-09-03

**Authors:** Huy Duc Vu, Phuong Tu Huynh, Junghwa Ryu, Ung Rae Kang, Sung Won Youn, Hongtae Kim, Hyun Jin Ahn, Kwankyu Park, Soon-Kyung Hwang, Young-Chae Chang, Yong Jig Lee, Hui Joong Lee, Jongmin Lee

**Affiliations:** 1 Department of Radiology, Daegu Catholic University School of Medicine, Daegu, Korea.; 2 Department of Anatomy, Daegu Catholic University School of Medicine, Daegu, Korea.; 3 Department of Pathology, Daegu Catholic University School of Medicine, Daegu, Korea.; 4 Department of Molecular Biology, Daegu Catholic University School of Medicine, Daegu, Korea.; 5 Department of Plastic Surgery, Daegu Catholic University School of Medicine, Daegu, Korea.; 6 Department of Radiology, Kyungpook National University School of Medicine, Daegu, Korea.

**Keywords:** magnetic iron oxide nanoparticles, melittin, intracranial arterial dolichoectasia, macrophages, extracellular matrix, matrix metalloproteinases

## Abstract

**Rationale:** In intracranial arterial dolichoectasia (IADE) development, the feedback loop between inflammatory cytokines and macrophages involves TNF-α and NF-κB signaling pathways and leads to subsequent MMP-9 activation and extracellular matrix (ECM) degeneration. In this proof-of-concept study, melittin-loaded L-arginine-coated iron oxide nanoparticle (MeLioN) was proposed as the protective measure of IADE formation for this macrophage-mediated inflammation and ECM degeneration.

**Methods:** IADE was created in 8-week-old C57BL/6J male mice by inducing hypertension and elastase injection into a basal cistern. Melittin was loaded on the surface of ION as a core-shell structure (hydrodynamic size, 202.4 nm; polydispersity index, 0.158). Treatment of MeLioN (2.5 mg/kg, five doses) started after the IADE induction, and the brain was harvested in the third week. In the healthy control, disease control, and MeLioN-treated group, the morphologic changes of the cerebral arterial wall were measured by diameter, thickness, and ECM composition. The expression level of MMP-9, CD68, MCP-1, TNF-α, and NF-κB was assessed from immunohistochemistry, polymerase chain reaction, and Western blot assay.

**Results:** MeLioN prevented morphologic changes of cerebral arterial wall related to IADE formation by restoring ECM alterations and suppressing MMP-9 expression. MeLioN inhibited MCP-1 expression and reduced CD68-positive macrophage recruitments into cerebral arterial walls. MeLioN blocked TNF-α activation and NF-κB signaling pathway. In the Sylvian cistern, co-localization was found between the CD68-positive macrophage infiltrations and the MeLioN distributions detected on Prussian Blue and T2* gradient-echo MRI, suggesting the role of macrophage harboring MeLioN.

**Conclusions:** The macrophage infiltration into the arterial wall plays a critical role in the MMP-9 secretion. MeLioN, designed for ION-mediated melittin delivery, effectively prevents IADE formation by suppressing macrophage-mediated inflammations and MMP activity. MeLioN can be a promising strategy preventing IADE development in high-risk populations.

## Introduction

Intracranial arterial dolichoectasia (IADE) is an arteriopathy characterized by dilated, elongated, and tortuous brain vasculature 1-4. In prevalence, the IADE was found up to 12% of patients presenting stroke while 0.1%-6.5% in the general population 1, 4-7. IADE can be asymptomatic but manifests as brain infarction 8,9, cerebral hemorrhage, subarachnoid hemorrhage 10, and compression syndrome of cranial nerve 11,12, brainstem, or third ventricle with hydrocephalus, resulting overall five-year fatality of 36.2% 4,7,13-15. Brain infarction can be a coincidental finding of IADE, but possibly caused by distorting and obstructing perforating arteries arising from the dolichoectatic artery or producing in-situ thrombosis and emboli 4,16. The increased vascular tortuosity in elderly patients is more likely to develop stroke of lacunar infarction pattern or small vessel occlusive disease, carrying a high risk of neurologic decline 9,17-19. Compressive symptoms manifest when dolichoectatic vertebrobasilar arteries exert pressure on cranial nerve roots and deforms the brainstem 20-24. Cranial neuropathies include ophthalmoplegia, nystagmus, hemifacial spasm, facial palsy, dizziness, tinnitus, hearing loss, dysarthria, trigeminal neuralgia 20-22 and/or diplopia 23. Cerebrospinal fluid (CSF) flow obstruction and hydrocephalus may occur when the basilar artery impinges on the third ventricle above the sellar diaphragm 25.

**Figure 1:**
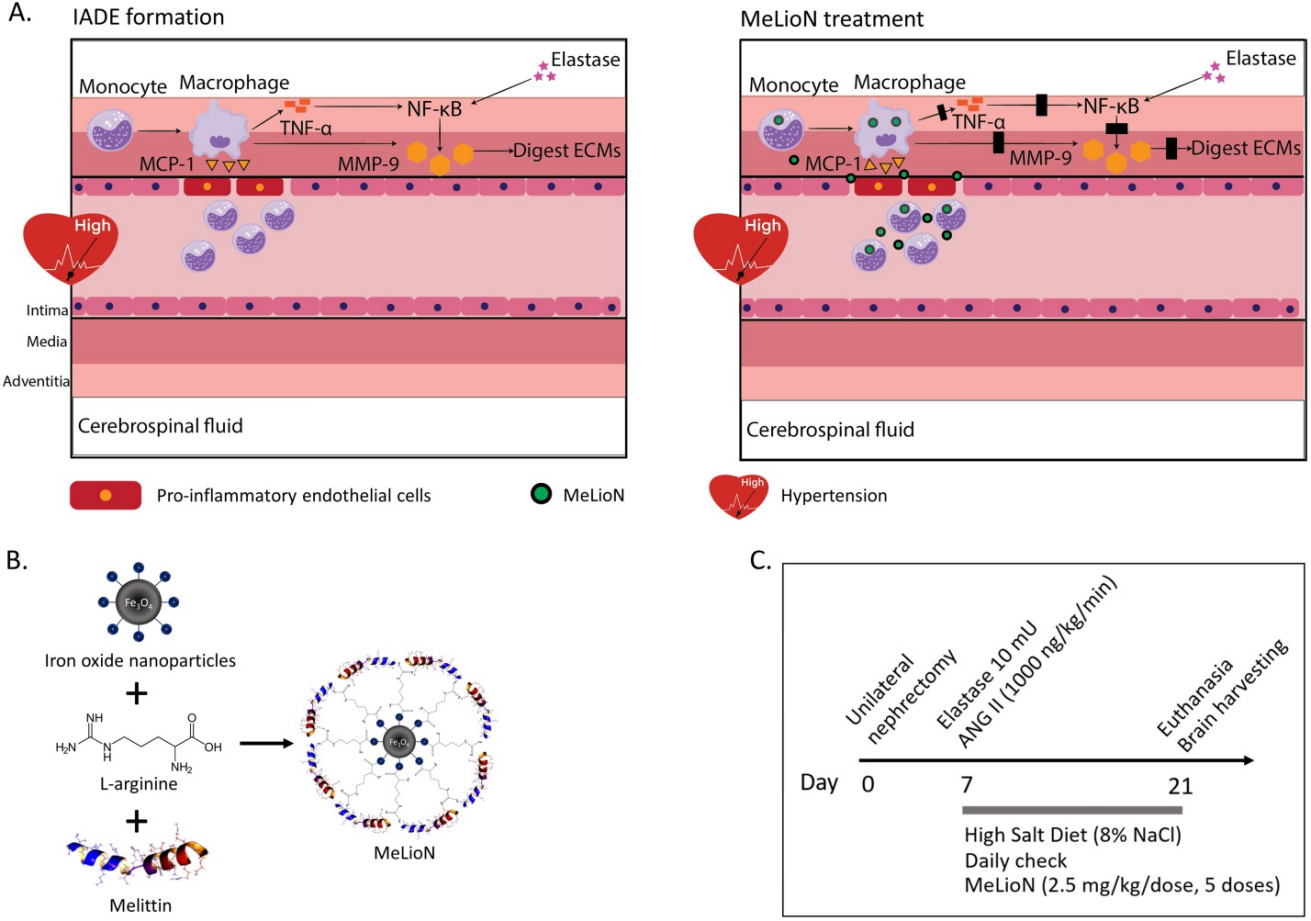
** Melittin-loaded L-arginine-coated iron oxide nanoparticle (MeLioN) prevents intracranial arterial dolichoectasia (IADE) formation by suppressing macrophage-mediated inflammation and pathologic extracellular matrix (ECM) remodeling. (A)** Inflammatory cascades underlying IADE and their blockage by MeLioN. Hemodynamic stress triggers endothelial cell dysfunction. In a mouse IADE model, hypertension was induced by unilateral nephrectomy, continuous angiotensin II infusion, and a high salt diet. Pro-inflammatory endothelial cells attract circulatory monocytes via inflammatory mediators such as MCP-1 production. The recruited monocytes at active inflammation foci differentiate into macrophages releasing MMP-9 through positive feedback and perturbation of the TNF-α and/or NF-κB signal pathway. Stereotaxic elastase injection into the cerebrospinal fluid space also causes MMP-9 activation and ECM digestion through NF-κB upregulation. Systemically administered MeLioNs are taken by monocytes and macrophages, which lead to attenuation of inflammation cascades (black block). MCP-1, monocyte chemoattractant protein-1; TNF-α, tumor necrosis factor-α; NF-κB, nuclear factor kappa-light-chain-enhancer of activated B cells, MMP-9, matrix metalloproteinase-9; ECM, extracellular matrix. **(B)** Design of MeLioN structure. Iron oxide nanoparticle was coated by L-arginine and melittin as a core-shell structure. **(C)** Admistration schedule of MeLioN. At day 0, hypertension or hemodynamic stress was induced by unilateral nephrectomy and high salt diet. At day 7, elastase was injected stereotaxically into right basal cistern and angiotensin II was continuously infused by subcutaneous osmotic pump implantation. The treatment started immediately after the IADE induction procedure by the dose of ION (0.1 mL, 1.25 mg/mL), MEL (1.0 mg/kg) and MeLioN (2.5 mg/kg) with five doses for every three days until the day 21 of brain harvest. At third weeks, brain was harvested.

Current management of IADE depends on clinical presentation and disease severity, including blood pressure control, antithrombotic regimens 26, endovascular procedures 27, and surgery 5,28. Surgical interventions for IADE are often associated with high mortality and morbidity because the dolichoectatic artery lacks a definite neck and is challenging to treat surgically 29. New non-invasive preventive strategies based on the pathophysiology underlying IADE may be an attractive alternative to improve the outcomes, and patients with dolichoectasia might benefit from early diagnosis. However, the efforts to investigate the pathophysiology underlying IADE have begun recently, although it has long been since reporting its clinical features 5,7. There have been no clinical trials to find effective treatment or guidelines for standard management established until now to delay its onset or mitigate the progression. This might be due to the lack of an appropriate animal model to understand pathophysiologic mechanisms and to find potential targets for therapy. Creating a proper animal model would shift the treatment paradigm by facilitating a better understanding of the mechanism and clinical translation of promising therapeutic agents. Dai et al. introduced the IADE model induced by elastase exposure similar to the abdominal aortic aneurysm (AAA) model 30-32. We describe the modified technique of creating and assessing cerebral arterial wall change induced by a single elastase injection into the basal cistern and induced hypertension. The histological findings demonstrated the rarefaction of the tunica media's elastic tissue and medial smooth muscle atrophy 33-35. We inferred that IADE development is closely linked to the hemodynamic stress-induced inflammation and ECM remodeling of the arterial wall. Since melittin peptide, the main component of honeybee venom, has been recently recognized in treating various chronic inflammatory diseases by inhibiting and modulating inflammatory cells and cytokines 36-40, we hypothesized that melittin would be effective likewise in suppressing inflammation and ECM remodeling affecting IADE development. However, melittin is a cationic cytolytic peptide that systemic administration causes adverse effects such as cell membrane pore formation and hemolysis 41. To address this shortcoming, we engineered a MeLioN by loading melittin on the surface of an L-arginine-coated iron oxide nanoparticle (LION) as a core-shell structure. As observed from various melittin nanoparticle formulations to neutralize its toxicity 42-47, we further tested the hypothesis that nanoparticle-mediated targeted delivery of melittin would circumvent this side effect and attenuate IADE development by targeting inflammatory cells and cytokines (Figure 1).

This study evaluated the protective effects of MeLioN on dolichoectasia formation in the experimentally induced murine model and investigated the underlying anti-inflammatory mechanism.

## Methods

### Reagents and Apparatus

The chemicals and reagents were purchased from Sigma-Aldrich (St. Louis, Missouri, USA), including ferrous chloride (FeCl_2_.4H_2_O), ferric chloride (FeCl_3_.6H_2_O), L-arginine, melittin (M2272), angiotensin II (A9525), elastase (E7885), and bromophenol blue dye. Alzet osmotic pumps (200 µL, 0.5 µL/hr) were supplied by Durect corporation (Cupertino, California, USA). The high salt diet containing 8% NaCl was purchased from Envigo (Indianapolis, Indiana, USA). The 10 µL model 701 syringe with 26G 2.0-inch point-style-3 Hamilton replacement needle was from Fisher Scientific (Hampton, New Hampshire, USA). The reagents and apparatus for western blot assay, Bradford and BCA protein assays, RNA extraction and cDNA synthesis kits and Trizol and SuperScript II RT were purchased from Thermo Fisher Scientific (Waltham, Massachusetts, USA). The antibodies were purchased from Abcam (Cambridge, UK) and Cell Signaling Technology (Danvers, Massachusetts, USA).

### Experimental Mouse Intracranial Arterial Dolichoectasia Induction

Animal care and all experimental procedures were approved and conducted in accordance with the guidelines of the Institutional Animal Care and Use Committee of the Catholic University of Daegu (Approval number: DCIAFCR-180727-09-Y). The IADE model was created in 8-week-old C57BL/6J male mice (Samtako, Osan, Korea) by inducing hypertension and elastase injection into the right basal cistern.

Animals were maintained on a 12-hour light-dark cycle with free access to normal rodent chow and water during one week of acclimation before the experiment. A total of 30 mice were randomly divided into 5 groups comprising of healthy control (H), disease control (D), three subgroups of different drug treatment including iron oxide nanoparticle (ION)-, free melittin (MEL)-, melittin-loaded L-arginine-coated iron oxide nanoparticle (MeLioN)-treated group. On the 0 day, the left side kidney was surgically removed under isoflurane anesthesia and all mice except healthy control began to be fed with a high-salt diet containing 8% sodium chloride for 21 days. At the first week, a single dose of elastase (10 μL of 1.0 U) was injected into the cerebrospinal fluid at right basal cistern (2.5 mm posterior, 1.0 mm lateral, 5.0 mm ventral to bregma, vertical needle insertion) at the rate of 0.2 μL/min under anesthesia, immediately followed by the subcutaneous implantation of an osmotic pump containing angiotensin II (1000 ng/kg/min). The treatment started after the dolichoectasia induction at the dose of ION (0.1 mL, 1.25 mg/mL), MEL (1.0 mg/kg) and MeLioN (2.5 mg/kg) with five doses for every three days. On third week, the mice were sacrificed by sequential cardiac perfusion at the left ventricle of 4 mL PBS, 4 mL 4% paraformaldehyde (PFA) and 4 mL solution of bromophenol blue dye dissolved in 10% (w/v) gelatine/PBS, respectively. Mouse brain tissues were harvested and immersed in 4% PFA in the refrigerator (4 °C) at least 24 hours before conducting histologic, gene and protein expression analysis.

### Histopathologic Analysis

After 24 hours of PFA fixation, specimens were paraffin-embedded. The blocks were sectioned into five slices based on the location of the circle of Willis cerebral arteries. Haematoxylin and Eosin served as basic stains for general morphology and analysis using sequential section-matched slices of brain tissue. Immunohistochemistry (IHC) was performed for the examination of elastin, collagen, and SMCs with corresponding staining kits such as Verhoeff Van Gieson, trichrome and α-SMA stains, respectively. Further special staining for pro-inflammatory mediators and metalloproteinase markers was conducted to examine the signal transduction. After mounting the tissue on the slide, the slide was scanned under an optical microscope at 40-time magnification to produce a histologic image. ImageJ software was used to select the region of interest (ROI) in the image. The area of ROI was measured and divided by the area of the whole cerebral artery to estimate the percentage of each component in the cerebral artery (Supplementary Figure 1). The morphologic changes of cerebral arterial wall related to dolichoectasia formation were measured by diameter, thickness, and ECM composition. For IHC of CD68, MCP-1, TNF-α and MMP-9, we proposed a scoring system classifying the expression level of these pro-inflammatory mediators in the cerebral arterial wall. In this scoring system, grade 0 was assigned to no marker expression in the cerebral artery, grade 1 to the marker expression less than, and grade 2 to more than surrounding a half of arterial wall circumference.

### Synthesis of MeLioN

Melittin solution was prepared by dissolving 5 mg of melittin powder in 1.0 mL PBS (pH=7.4). For the MEL-treated group, 0.125 mg/mL melittin solution was prepared from the 5.0 mg/mL concentrated solution based on the suggested dose at 1 mg/kg. The other 0.36 mM (~1.0 mg/mL) melittin solution was prepared to load onto nanoparticles' surface. Melittin solution remains stable at 4 °C in the refrigerator for eight weeks 46. L-arginine-coated-ION (LION) was synthesized by the chemical co-precipitation under the nitrogen gas. Ferrous chloride (FeCl_2_.4H_2_O) and ferric chloride (FeCl_3_.6H_2_O) with the molar ratio of 1:2 were dissolved in 20 mL degassed ultrapure water mixed well with 20 mL of 0.07 % L-arginine solution. 7 mL of 25 % NH_4_OH was drop-wise added into the reaction vessel with vigorous stirring at 1000 rpm at 80 °C for 1 hour until the complete precipitation. LION samples were rinsed three times respectively with water and ethanol and dried in an oven at 60 °C for 24 hours. A 2.5 mg/kg of melittin was loaded on the LION surface as the safe dose suggested by Soman et al. 42. The 0.5 mL of 0.36 mM melittin solution was added to the 0.5 mL of 2.5 mg/mL LION suspension in a 15 mL centrifuged tube. The mixture was gently agitated at 4 °C for 48 hours. After completing the loading process, samples were stood for 10 minutes before separating the supernatant and pellet by centrifugation (200 g, 10 min). The unbound melittin in the supernatant was carefully removed and stored in another container to determine loading efficacy. The soft pellet was then washed three times and re-dispersed in 1 mL deionized water.

### Nanoparticles Characterization

**Figure 2:**
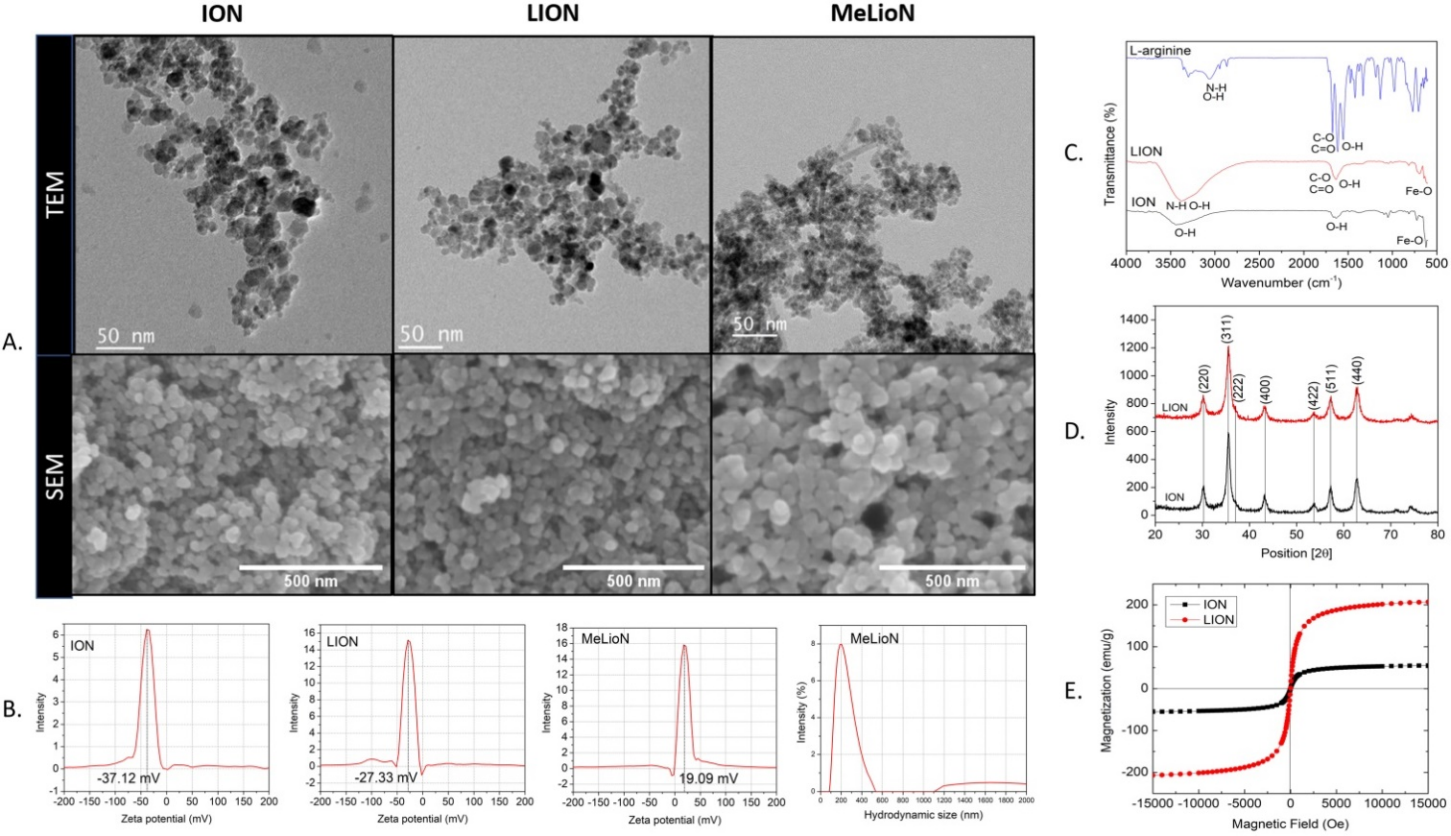
** Characterizations of bare (ION; Fe_3_O_4_), L-arginine coated (LION), and melittin-loaded L-arginine-coated iron oxide nanoparticle (MeLioN). (A)** Scanning electron microscopy (SEM) and transmission electron microscopy (TEM) images showed grossly spherical shapes and good dispersion of small-sized nanoparticles (10-30 nm). **(B)** Dynamic light scattering analysis for zeta potential and hydrodynamic size. Zeta potential of ION, LION, and MeLioN was -37.12 mV, -27.33 mV, and 19.09 mV, respectively. The hydrodynamic size of MeLioN was approximately 202.4 nm, polydispersity index, 0.158. **(C)** Fourier-transform infrared spectroscopy (FTIR). The L-arginine coating layer was confirmed on the surface of ION based on the comparison of the FTIR spectra of pure L-arginine, ION and LION. **(D)** X-ray diffraction (XRD) analysis. No difference was found in the pattern of XRD spectra between ION and LION. XRD pattern of magnetite demonstrates the featured 220, 311, 400, 422, 511, and 440 peaks of Fe_3_O_4_ crystals with a cubic spinel structure at ~2θ of 30°, 35°, 43°, 53°, 57°, 62° and 74°. The average crystallite size of ION and LION was 11.11
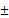
2.16 nm and 8.31
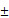
2.07 nm, respectively. **(E)** Hysteresis curve revealing magnetization of ION (55.28 emu/g) and LION (207.05 emu/g) at 10 kOe.

The microscopic images of bare (ION; Fe_3_O_4_), LION, and MeLioN were obtained from Field Emission Transmission Electron Microscope (FETEM; JEM2100F) at 200 kV, and Field Emission Scanning Electron Microscope (FESEM) (Hitachi HI-9116-0002) at 5kV. SEM and TEM images showed spherical shapes and good dispersion of small-sized nanoparticles (20-40 nm) (Figure 2A). Dynamic Light Scattering instrument (DLS; Particle and Pore Size Analysis System CJ102) measured the zeta potential of nanoparticle surface charge and hydrodynamic size. Zeta potential of ION, LION, and MeLioN was -37.12 mV, -27.33 mV, and 19.09 mV, respectively (Figure 2B). The hydrodynamic size of MeLioN dispersed in deionized water was approximately 202.4 nm with the polydispersity index of 0.158 (Figure 2B), which was stable over two weeks measurement (Supplementary Figure 2). Fourier Transform Infrared (FT-IR) spectroscopy was conducted using FT-UV-VIS-IR Spectroscopic Imaging Microscope Vertex 80 with the wavenumber ranging from 600-4000 cm^-1^ using attenuated total reflection method. The L-arginine coating layer was confirmed on the surface of ION by comparing the IR spectra of pure L-arginine, LION, and ION. No difference of crystalline structure was found in the pattern of X-Ray diffraction (XRD; Multi Purpose X-Ray Diffractometer NE03) spectra between ION and LION. XRD pattern of magnetite demonstrates the featured 220, 311, 400, 422, 511, and 440 peaks of Fe_3_O_4_ crystals with a cubic spinel structure at ~2θ of 30°, 35°, 43°, 53°, 57°, 62°, and 74°. The average crystallite size was 6.29±1.17 nm calculated from Scherrer equation. Vibrating sample magnetometer obtained hysteresis curve revealing magnetization of ION (55.28 emu/g) and LION (207.05 emu/g) at 10 kOe.

### Melittin-loading Capacity of MeLioN

The amount of melittin loaded in the MeLioN was measured by tryptophan-sensitive fluorescence assay and the Bradford method (Supplementary Figure 3). The amount of loaded melittin was calculated based on the calibration relating melittin concentration to the fluorescence intensity. Melittin has a tryptophan residue with the excitation and emission wavelength at 280 nm and 350 nm on the fluorescence intensity curve. In the Bradford method, the concentration of loaded melittin in MeLioN was measured by mixing the known amount of melittin (0.5 mL, 0.36 mM) and LION (0.5 mL, 2.5 mg/mL) into the centrifuge tube. Centrifuge separated the supernatant and the pellet, and the unbound melittin in the supernatant was analyzed by reacting with Coomassie Brilliant Blue G-250 at room temperature for 5 min. The concentration of melittin is calibrated from the absorbance of the wavelength at 595 nm. The concentration of melittin contained within MeLioN (*C_MEL/MeLioN_*) was calculated by subtracting the free melittin unbound to LION (*C_free MEL/supernatant_*) from the total known amount of melittin (*C_MEL/initially added_*); *C_MEL/MeLioN_* = *C_MEL/initially added_* - *C_free/supernatant_*. The amount of melittin within MeLioN was measured as 246.5±0.59 µg/mL by tryptophan-fluorescence and 256.79±1.95 µg/mL by the Bradford method, respectively.

### *In vitro* Melittin Release

MeLioN was dispersed in PBS, and the supernatant was collected at given time intervals (2, 4, 8, 24, and 48 h) at different temperatures at 37 °C and 4 °C. For the collecting melittin, each tube was centrifuged at 10,000 rpm for 10 min. The release amount of melittin was determined by a microplate reader at 562 nm (Molecular Devices, San Jose, CA, USA) using a standard calibration curve of bovine serum albumin standard.

### Quantitative Real Time-Polymerase Chain Reaction (RT-PCR)

Total mRNA was isolated using TRIzol reagent (Thermo Fisher) from the left lower quadrant portion of the mouse brain tissue containing the circle of Willis. This was followed by a reverse transcription reaction to produce cDNA templates using Superscript IV Reverse Transcriptase and random primers packaged in a high-throughput kit. RT-PCR was performed in a Mini Opticon system from Bio-Rad with Takara SYBR Green. The 20 µL PCR mixtures contained less than 100 ng of cDNA templates and 0.4 µM each of the forward and reverse primers (Supplementary Table 1). The samples were denatured at 95 °C for 1 min, followed by 41 cycles of annealing and extension at 95 °C for 5 s, annealing temperature for 15 s, and 72 °C for 15 s. The measured expression values of each target gene were normalized to the expression values of each endogenous gene by using glyceraldehyde 3-phosphate dehydrogenase (GAPDH).

### Western Blot Assay

The right lower quadrant of the mouse brain tissue containing the circle of Willis was snap-frozen in liquid nitrogen and stored at -80 °C. After extraction using a radioimmunoprecipitation assay buffer, the optical density suggesting protein sample concentration was measured at 562 nm through BCA assay (Pierce BCA protein assay kit; Thermo Fisher). The protein samples of each study group were normalized as the concentration of 1 µg/µL before loading onto the gel. The protein samples were then separated on the precast gradient polyacrylamide gels (Bolt^TM^ 4-12% Bis-Tris Plus Gel) and transferred to polyvinylidene difluoride (PVDF) membrane packaged within a ready-to-use iBlot®2 Transfer Stack using the Bolt^TM^ Mini Gel Tank and iBlot®2 Transfer Device (Invitrogen). After transfer, the membrane was blocked in 5% skim milk (Difco) in 1 hour. The blocked membrane was then gently washed with 1X Tris-Buffered Saline (TBS) - Tween in 2 min and probed with a primary antibody at room temperature in 2 hours. Following three-time washing steps in 30 min, the membrane was incubated with a horseradish peroxidase-conjugated secondary antibody at room temperature in 1 hour. Following a repeat of washing steps, the membrane was added to an enhanced chemiluminescence detection reagent (Super Signal® West Femto) for 1 min. Signal intensity was measured with an image analyzer (ChemiDoc^TM^ XRS+; Bio-Rad Laboratories, Hercules, CA, USA). Pro-inflammatory cytokines were separated by their weight and visualized as the white blot in the black background.

### Biosafety and Brain Clearance of MeLioN in the Healthy Mice

To assess the biosafety profiles of MeLioN, blood samples and major organs specimen of healthy mice were obtained at the one day (n=4), one week (n=4), and one month (n=4) after MeLioN administration via a tail vein, respectively. The serum levels of AST, ALT, bilirubin, BUN, and creatinine were assessed for the surveillance of the damage to the liver and kidney. The complete blood counts were monitored to find any hematologic abnormality. The Hematoxylin and Eosin stain was performed in the brain, heart, liver, kidney, spleen, and lung specimens.

To assess the clearance of MeLioN from the healthy mice brain, inductively coupled plasma-mass spectrometry (ICP-MS) determined the concentration of MeLioN in the brain by the concentration of total iron content at the one-day (n=4), one-week (n=4), and one-month (n=4) groups after MeLioN administration.

### Cell Viability Test

The cell viability of RAW 264.7 cells was determined by MTT assay (ab211091; Abcam, Cambridge, UK). The cells were seeded in a 96-well culture plate at 5.0 × 10^3^ cells per well and pre-incubated in 24 h. After pre-incubation, the cells were treated with free melittin (0.1, 0.5, 1, 2 and 4 µg/mL) and MeLioN (equivalent dosages) for 12 h or 24 h. After treatment, 50 µL of MTT reagent [3-(4,5-dimethylthiazol-2-yl)-2,5-diphenyltetrazolium bromide] was added to each well, and the cells were incubated for additional 3 h at 37 °C. After incubation, 150 µL of MTT solvent was added into each well to fully dissolve the MTT formazan. The cell viability values were then measured by absorbance at 590 nm using a microplate reader. Each experiment was done in triplicate. The relative cell viability (%) was determined by normalizing the absorbance of the test sample to the absorbance of the control sample as presented in the below formula:


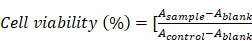
]

where *A_sample_* is the absorbance of the test sample, *A_control_* is the absorbance of the control sample, and *A_blank_* is the absorbance of the blank sample.

### Hemolytic Activity of MeLioN and Free Melittin

Mouse red blood cells were washed with phosphate-buffered saline (PBS), and the supernatant was cleared by centrifugation at 1000 ×g for 5 min. A 200 µL of blood was taken from the bottom of the tube and added to the 9.8 mL of PBS. Using PBS as blank and 0.025% Triton X-100 as the positive control, either free melittin or MeLioN (1, 5, 10, 20, 50 µg) were added into 200 µL of diluted blood in different tubes. The tubes were incubated for 1 h at 37 °C. Then, the mixture was centrifuged at 1000 × g for 15 min. A 100 µL supernatant was transferred to a 96-well plate and detected by measuring the optical density (OD) at 414 nm (Molecular Devices, San Jose, CA, USA). The percentage of hemolysis was calculated as follows: hemolysis (%) = (A_sample_ - A_PBS_)/(A_triton_ - A_PBS_) × 100, where A_sample_ is the absorbance of the sample, A_triton_ is the absorbance of the triton-treated sample, and A_PBS_ is the absorbance of the PBS. A standard curve was plotted by measuring the OD of diluted supernatant of Triton X-100 hemolyzed blood (0, 12.5, 25, 50, and 100%). The hemolysis of nanocomposites was determined using the standard curve.

### Statistical Analysis

Statistical analysis was performed by using a one-way analysis of variance (ANOVA) test followed by Dunnett's post hoc comparison for multiple-group comparison. Student t-test was used for two-group comparison in MTT assay. χ^2^ test was used for proportion comparison in the expression of MCP-1, CD68, TNF-α and NF-κB. The data of diameter and thickness was presented as mean ± standard of deviation (SD). The other data was presented as the mean ± standard error of the mean (SEM). All statistical analyses and graphics were conducted by using Medcalc software (version 18.2.1) and GraphPad Prism 9 software. Differences with *p-*value < 0.05 were considered statistically significant.

## Results

### MeLioN Showed Favorable Safety Profiles and Brain Clearance in the Healthy Mice

One week after MeLioN administration, serum BUN level was elevated, but serum AST and ALT levels were not different from those of the healthy mice (Supplementary Figure 5). However, there was no significant difference of AST, ALT, and BUN level between the control and one-month groups after MeLioN administration (AST, 168.00±23.71 mU/mL versus 149.75±39.44 mU/mL, ns; ALT, 33.33±3.33 mU/mL versus 29.75±0.85 mU/mL, ns; BUN. 16.5±1.32 versus 19.75±0.63 mg/dL, ns). The creatinine and bilirubin levels were not detected in all groups (creatinine, < 0.2 mg/dL; bilirubin, < 0.1 mg/dL).

In the complete blood counts, the RBC, WBC, and platelets were not significantly different between control and one-month groups (RBC, 8.9±0.14 10^6^/µL versus 9.57±0.04 10^6^/µL, ns WBC (1.94±0.29 10^3^/µL versus 1.75±0.36 10^3^/µL, ns), and platelets (219.00±39.21 10^3^/µL versus 398.25±94.11 10^3^/µL, ns). The hemoglobin level was decreased in one week (13.15±0.26 g/dL) but within the normal range at one months later (14.35±0.29 g/dL versus 13.60±0.14 g/dL, ns).

There were no inflammatory cell infiltrations, necrosis, or fibrotic changes in the brain, heart, liver, kidney, spleen, and lung tissues on the control, 24 h-, one-week-, and one-month-post injected groups (Supplementary Figure 6).

The total iron content in the brain of the 24 h-post injected MeLioN group was significantly higher than that of the control group (Supplementary Figure 7; 2.57±0.96 mg Fe/g tissue versus 0.38±0.27 mg Fe /g tissue, p < 0.05), while the iron content in the brain of the 1-month-post injected MeLioN group was not significantly different from that of the control group (0.76±0.32 mg Fe/g tissue versus 0.38±0.27 mg Fe/g tissue, ns).

### MeLioN Prevented Morphologic Changes of Cerebral Arterial Wall related to Intracranial Arterial Dolichoectasia Formation

**Figure 3:**
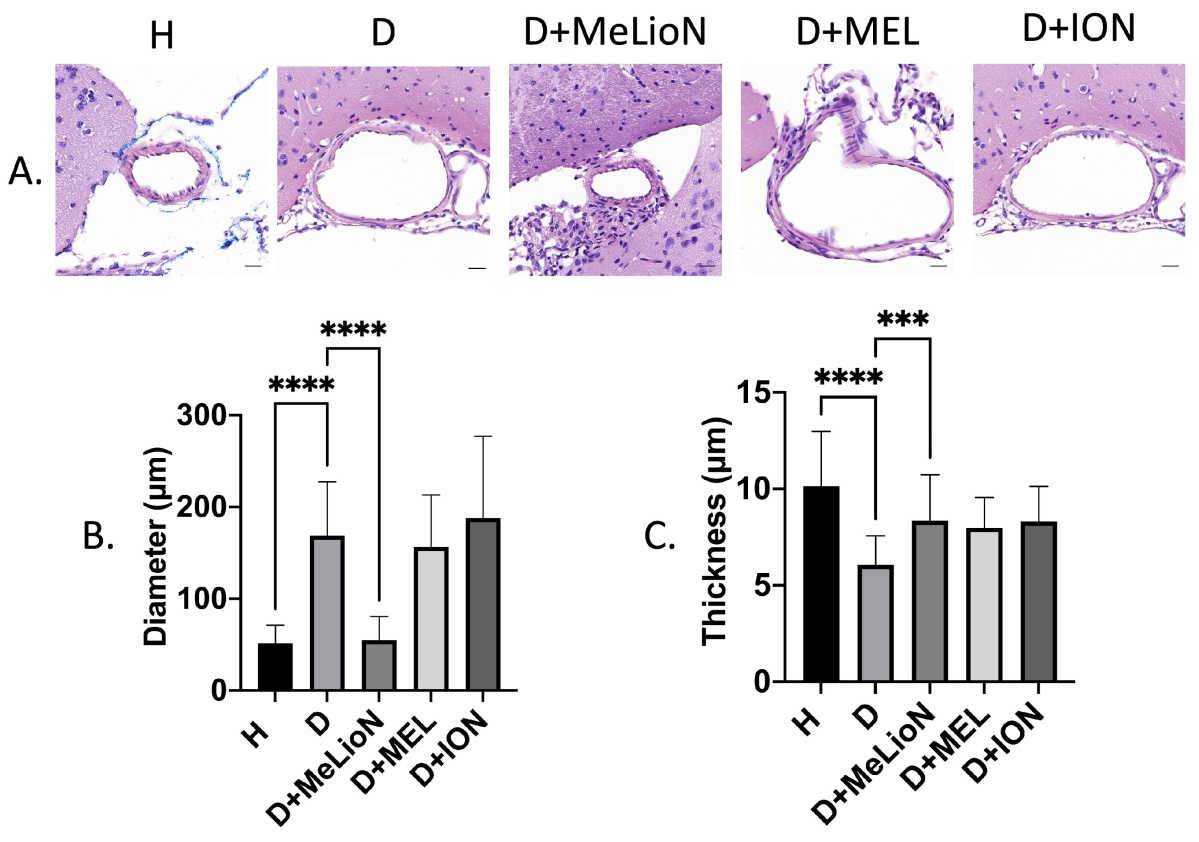
** Inhibitory effects of melittin-loaded L-arginine-coated iron oxide nanoparticle (MeLioN) on morphologic alterations of mouse cerebral arterial walls 3 weeks after experimental dolichoectasia induction. (A)** Hematoxylin and Eosin stain of H (healthy control), D (disease control), ION (iron oxide nanoparticle), MEL- (free melittin), and MeLioN-treated group. **(B)** Diameter. In the disease control, the cerebral arterial diameter was significantly higher than that of healthy control (Dunnett's post hoc, *p*<0.0001; n=6). Treatment with MeLioN reduced the cerebral arterial diameter comparable to that of healthy control (ns). **(C)** Thickness. In disease control, the cerebral arterial wall thickness was significantly lower than that of healthy control (*p*<0.0001). Treatment with MeLioN increased the cerebral arterial thickness significantly higher than disease control (*p*<0.001). Each MEL group or ION group showed a significantly larger diameter than MeLioN group (*p*<0.0001; *p*<0.0001) but did not show any difference in arterial wall thickness as compared to that of MeLioN group (ns; ns), respectively. ****, *p*<0.0001; ***, *p*<0.001. The data were expressed as mean±SD. Scale bar: 20 µm.

In the disease control, the cerebral arterial diameter was significantly larger than that of healthy control (168.7±58.7 μm versus 51.4±19.6 μm; Dunnett's post hoc, *p*<0.0001; n=6). Treatment with MeLioN reduced the cerebral arterial diameter (54.9±25.6 μm) comparable to that of healthy control (ns; Figure 3). In the disease control, the cerebral arterial wall thickness was significantly lower than that of healthy control (6.1±1.5 μm versus 10.2±2.8, *p*<0.0001). Treatment with MeLioN increased the cerebral arterial thickness (8.4±2.4 μm) significantly higher than that of disease control (*p*<0.001). Each MEL-treated group or ION-treated group showed a significantly larger diameter than the MeLioN-treated group (*p*<0.0001; *p*<0.0001) but did not show any difference in arterial wall thickness as compared to that of the MeLioN group (ns; ns), respectively.

### MeLioN Restored ECM Alterations in Cerebral Arterial Walls related to Intracranial Arterial Dolichoectasia Formation

**Figure 4:**
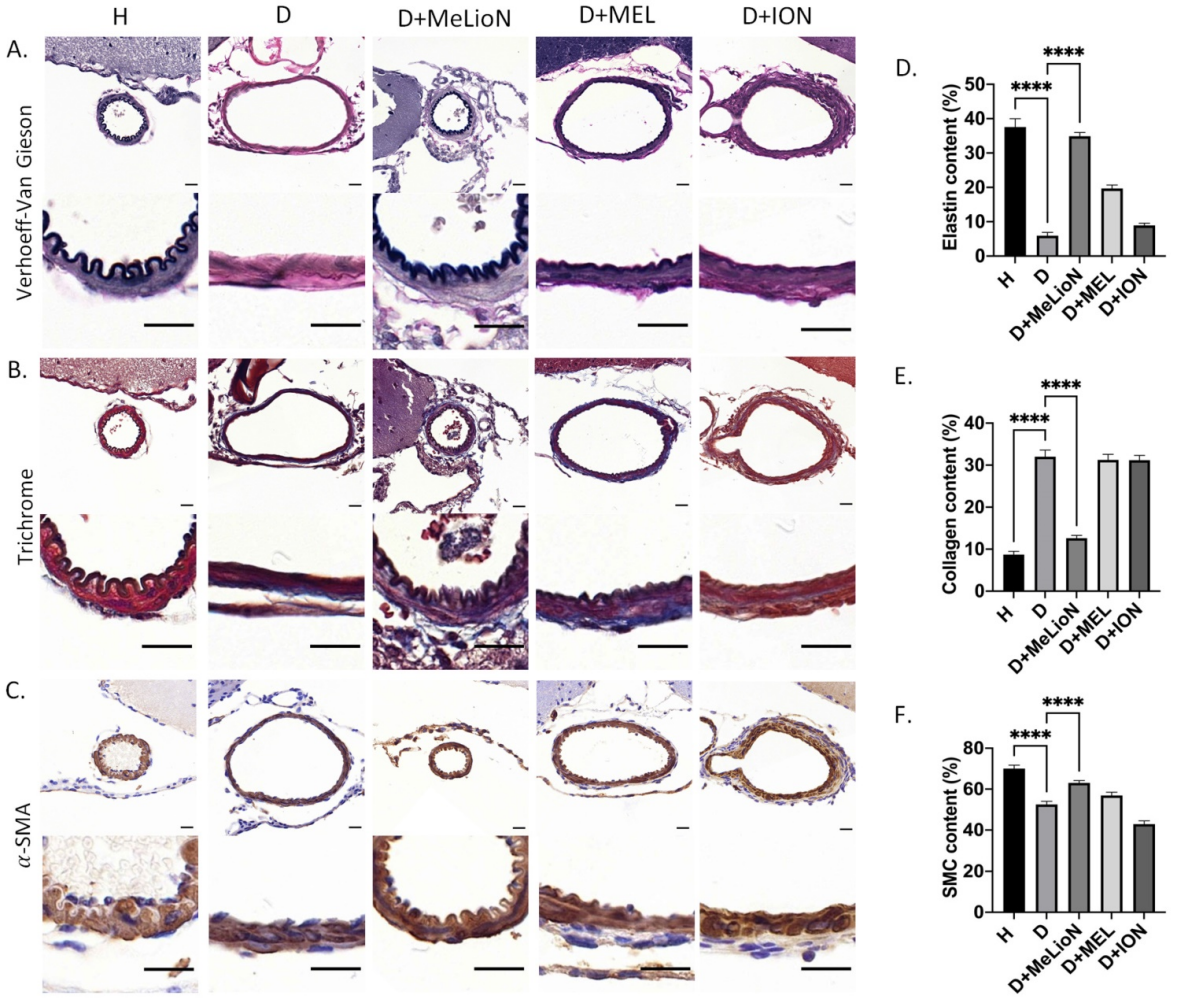
** Protective effect of melittin-loaded L-arginine-coated iron oxide nanoparticle (MeLioN) on the pathologic extracellular matrix (ECM) remodelling of mouse cerebral arterial walls 3 weeks after experimental dolichoectasia induction. (A)** Elastica van Gieson,** (B)** Trichrome, **(C)** α-SMA stain of H (healthy control), D (disease control), ION (iron oxide nanoparticle), MEL- (free melittin), and MeLioN-treated group. In disease control, there found a loss of elastin lamina, over-production of collagen, and smooth muscle cell (SMC) apoptosis, while in MeLioN-treated group, these ECM alterations were alleviated in arterial walls. Scale bar: 20 µm. **(D, E, F)** In disease control, the elastin and SMC content were significantly decreased as compared to that of healthy control (*p*<0.0001; *p*<0.0001). MeLioN treatment restored the elastin content to comparable to that of healthy control (*ns*), and the SMC content significantly higher than that of disease control (*p*<0.0001). The MEL group or ION group showed significantly lower content of elastin (*p*<0.0001; *p*<0.0001) and SMC (*p*<0.01; *p*<0.0001) than that of MeLioN group, respectively. The collagen content was significantly increased in disease control, compared to that of healthy control (*p*<0.0001). MeLioN treatment reduced the collagen content (12.6%) to lower than that of disease control (*p*<0.0001). The MEL group or ION group showed significantly higher content of collagen (*p*<0.0001; *p*<0.0001) than that of MeLioN-treated group, respectively.

The elastin and smooth muscle cell (SMC) content was significantly decreased in disease control compared to that of healthy control (5.9±3.7% versus 37.6±8.8%, *p*<0.0001; 52.5±8.8% versus 70.0±9.5%, *p*<0.0001), respectively. MeLioN treatment restored the elastin content (34.9±6.2%) to comparable to that of healthy control (ns), and the SMC content (63.0±8.4%) significantly higher than that of disease control (*p*<0.0001; Figure 4). The collagen content was significantly increased in disease control than that of healthy control (32.0±5.5% versus 8.7±3.8%, *p*<0.0001). MeLioN treatment reduced the collagen content (12.6±5.1%) to lower than that of disease control (*p*<0.0001). The MEL group or ION group showed significantly lower elastin content (*p*<0.0001; *p*<0.0001) and SMC (*p*<0.01; *p*<0.0001) than that of the MeLioN-treated group, respectively. The MEL-treated group or ION-treated group showed significantly higher collagen content (*p*<0.0001; *p*<0.0001) than that of the MeLioN-treated group, respectively.

### MeLioN Suppressed MMP-9 Expression in Cerebral Arterial Walls

**Figure 5:**
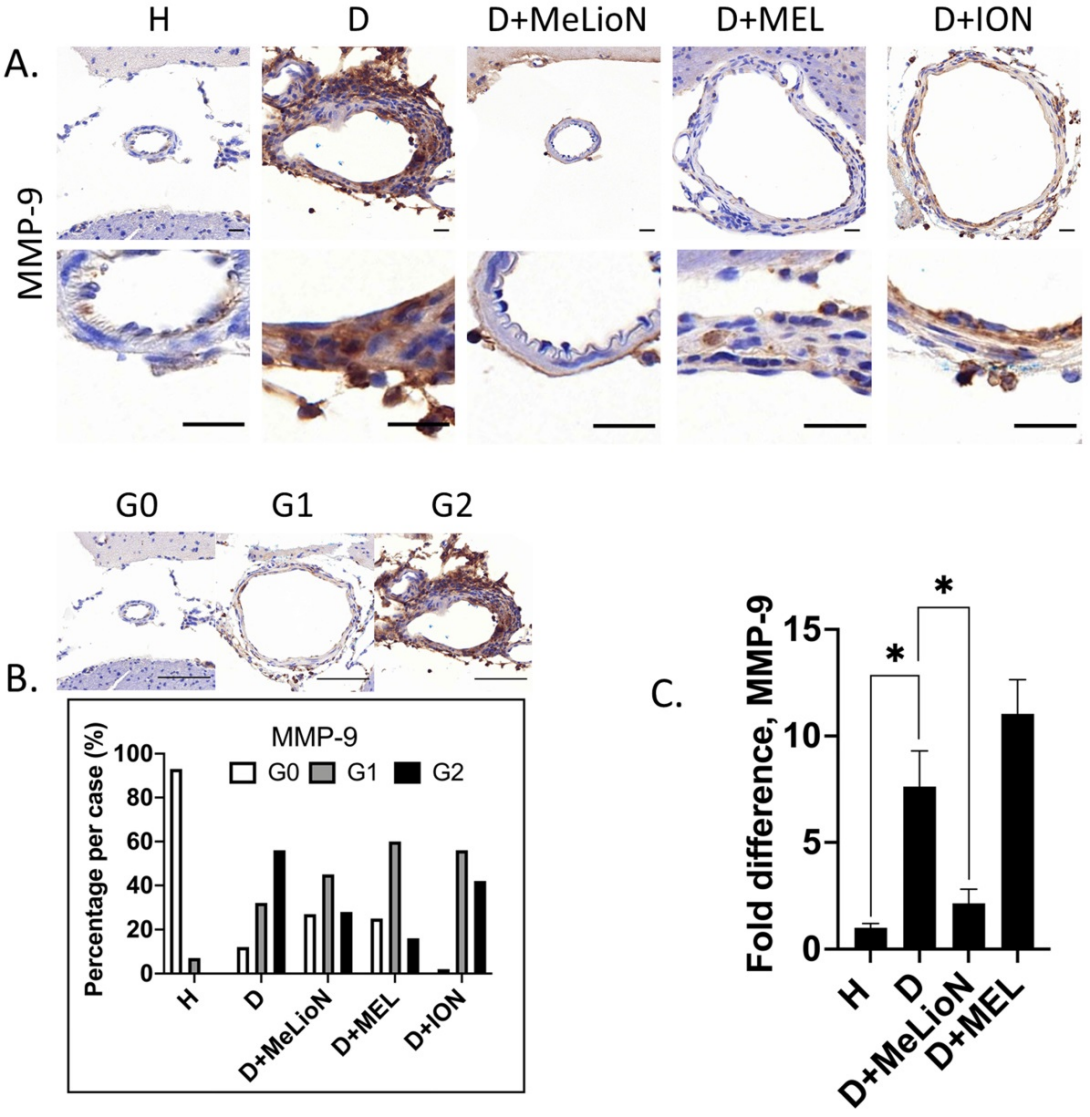
** Matrix metalloproteinase (MMP-9) activity in the experimentally induced mouse cerebral arterial wall. (A)** MMP-9 immunohistochemistry statin of H (healthy control), D (disease control), ION (iron oxide nanoparticle), MEL (free melittin), and MeLioN (melittin-loaded L-arginine-coated iron oxide nanoparticle) group. MeLioN treatment attenuated MMP-9 expression in the arterial wall. Scale bar: 20 µm in A and 50 µm in B. **(B)** MMP-9 activity grading based on immunohistochemistry. Grade 0, absence; Grade 1, presence of less than half of the arterial wall circumference; Grade 2, presence of more than half of the arterial wall circumference. In disease control, the percentile of MMP-9 grade 0 and grade 2 was significantly lower and higher than that of healthy control (*p*<0.0001; *p*<0.0001), respectively. The MeLioN-treated group exhibited decreased expression of MMP-9, with a significantly higher and lower percentile of grade 0 and grade 2 than that of disease control (*p*<0.05; *p*<0.001), respectively. The MEL group did not show any difference in MMP-9 grade 0 proportion (ns) and grade 2 proportion (ns) as compared to the MeLioN group, respectively. ION group showed a significantly lower proportion of MMP-9 grade 0 (*p*<0.001) but no statistical difference in MMP-9 grade 2 proportion as compared to MeLioN group (ns), respectively. **(C)** Real-time polymerase chain reaction for MMP-9. In disease control, MMP-9 mRNA expression was 8 times up-regulated on 3 weeks after dolichoectasia induction compared to healthy control (*p*<0.05). In the MeLioN-treated group, MMP-9 mRNA expression levels reduced to be 25% of disease control (*p*<0.05).

The effects of MeLioN on the MMP-9 expression in mice cerebral arterial walls after three weeks of IADE induction were evaluated by IHC grading and RT-PCR. IHC analysis revealed that treatment with MeLioN attenuated MMP-9 expression in arterial walls (Figure 5). In term of MMP-9 expression in the arterial wall, the grade 0 and grade 2 proportion of disease control was significantly lower and higher than healthy control (11.7% versus 92.9%, *p*<0.0001; 56.4% versus 0.0%, *p*<0.0001; χ^2^ test), respectively. The MeLioN-treated group exhibited decreased expression of MMP-9, with significantly higher grade 0 and lower grade 2 proportion than disease control (26.7% versus 11.7% %, *p*<0.05; 28.3% versus 56.4%, *p*<0.001), respectively. The MEL group did not show any difference in MMP-9 grade 0 proportion (ns) and grade 2 proportion (ns) as compared to the MeLioN group, respectively. The ION group showed a significantly lower proportion of MMP-9 grade 0 (*p*<0.001) but no statistical difference in MMP-9 grade 2 proportion as compared to the MeLioN group (ns), respectively. In quantitative RT-PCR analysis, MMP-9 mRNA expression was 7.6 times up-regulated three weeks after IADE induction compared to healthy control (*p*<0.05). However, the MMP-9 mRNA expression of MeLioN-treated group reduced to 28.9% of disease control (*p*<0.05). In Western blot, the MMP-9 band of disease control was more intense than healthy control. The MMP-9 bands were indistinct in both MeLioN- and MEL-treated groups (Supplementary Figure 4).

### MeLioN Inhibited MCP-1 Expression and Reduced subsequent CD68-positive Macrophage Recruitments into Cerebral Arterial Walls

**Figure 6:**
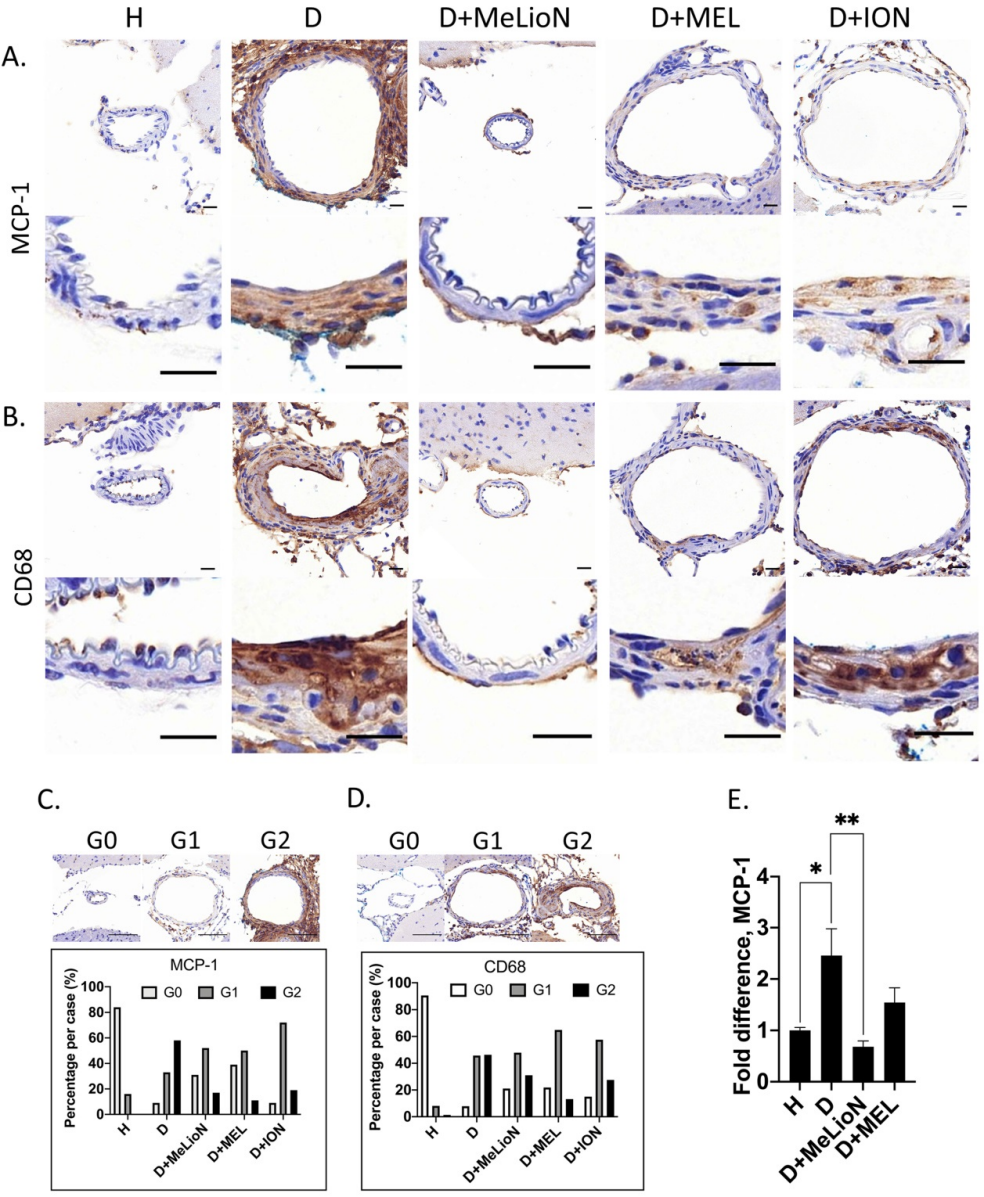
** Monocyte chemoattractant protein (MCP-1) expression and CD68-positive macrophage infiltration in the experimentally induced mouse cerebral arterial wall.** Immunohistochemistry showed that MeLioN treatment attenuated MCP-1 expression **(A)** and CD68-positive macrophage infiltration **(B)** in arterial walls. H, healthy control; D, disease control, ION, iron oxide nanoparticle-treated group; MEL, free melittin-treated group; MeLioN, melittin-loaded L-arginine-coated iron oxide nanoparticle-treated group. Scale bar: 20 µm in A and B, 50 µm in C and D. **(C)** Grading of MCP-1 expression. Grade 0, absence; Grade 1, presence of less than half of arterial wall circumference; Grade 2, presence of more than half of arterial wall circumference. In disease control, the percentile of MCP-1 grade 0 and grade 2 was significantly lower and higher than that of healthy control (*p*<0.0001; *p*<0.0001; 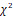 test). The MeLioN-treated group exhibited decreased expression of MCP-1, with significantly higher grade 0 and lower grade 2 proportion than disease control (*p*<0.001; *p*<0.0001). **(D)** Grading of CD68-positive macrophage. In disease control, the percentile of macrophage grade 0 and grade 2 was significantly lower and higher than healthy control (*p*<0.0001; *p*<0.0001). The MeLioN-treated group reduced CD68-positive macrophage infiltration, with significantly higher percentile of grade 0 and lower percentile of grade 2 than that of disease control (*p*<0.01; *p*<0.05). **(E)** Real-time polymerase chain reaction. In disease control, MCP-1 mRNA expression was 2.5 times up-regulated compared to healthy control (*, *p*<0.05). In the MeLioN-treated group, MCP-1 mRNA expression levels were lower than disease control (**, *p*<0.01) but comparable to healthy control (ns).

By using IHC grade and RT-PCR, the effects of MeLioN were evaluated on the MCP-1 expression and macrophage infiltration into mouse cerebral arterial wall at three weeks of IADE induction (Figure 6). IHC analysis revealed that MeLioN treatment attenuated MCP-1 expression of and CD68-positive macrophage infiltration into arterial walls. In term of MCP-1 expression in the arterial wall, the grade 0 and grade 2 proportion of disease control was significantly lower and higher than healthy control (9.1% versus 84.1%, *p*<0.0001; 58.0% versus 0.0%, *p*<0.0001; 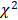 test), respectively. MeLioN-treated group reduced expression of MCP-1, with significantly higher grade 0 and lower grade 2 proportion than disease control (31.3% versus 9.1%, *p*<0.001; 17.2% versus 58.0%, *p*<0.0001), respectively. The MEL group did not show any difference in MCP-1 grade 0 proportion (ns) and grade 2 proportion *(*ns*)* as compared to the MeLioN group, respectively. The ION group showed a significantly lower proportion of MCP-1 grade 0 (*p*<0.01) but no statistical difference in MCP1 grade 2 proportion as compared to the MeLioN group (ns), respectively.

In term of CD68-positive macrophage infiltration into the arterial wall, the grade 0 and grade 2 proportion of disease control was significantly lower and higher than healthy control (7.9% versus 90.5%, *p*<0.0001; 46.3% versus 1.4%, *p*<0.0001), respectively. MeLioN-treated group reduced CD68-positive macrophage infiltration, with significantly higher grade 0 and lower grade 2 proportion than disease control (21.1% versus 7.9%, *p*<0.01; 31.0% versus 46.3%, *p*<0.05), respectively. The MEL group did not show any difference in CD68-positive macrophage grade 0 proportion (ns) but showed lower CD68-positive macrophage grade 2 proportion (13.2% versus 31.0%, *p*<0.01) as compared to MeLioN group, respectively. The ION group did not show any statistical difference in proportion of CD68-positive macrophage grade 0 (ns) and grade 2 proportion (ns) as compared to MeLioN group, respectively. In quantitative PCR analysis, MCP-1 mRNA expression was 2.5 times up-regulated three weeks after IADE induction compared to healthy control (*p*<0.05). In the MeLioN-treated group, MCP-1 mRNA expression levels were lower than disease control (**, *p*<0.01) but comparable to healthy control (ns). In Western blot, the MCP-1 band was intense in disease control as compared to healthy control. The MCP-1 band became indistinct in both MeLioN- and MEL-treated group.

### MeLioN blocked TNF-α activation and NF-κB Signaling Pathway

**Figure 7:**
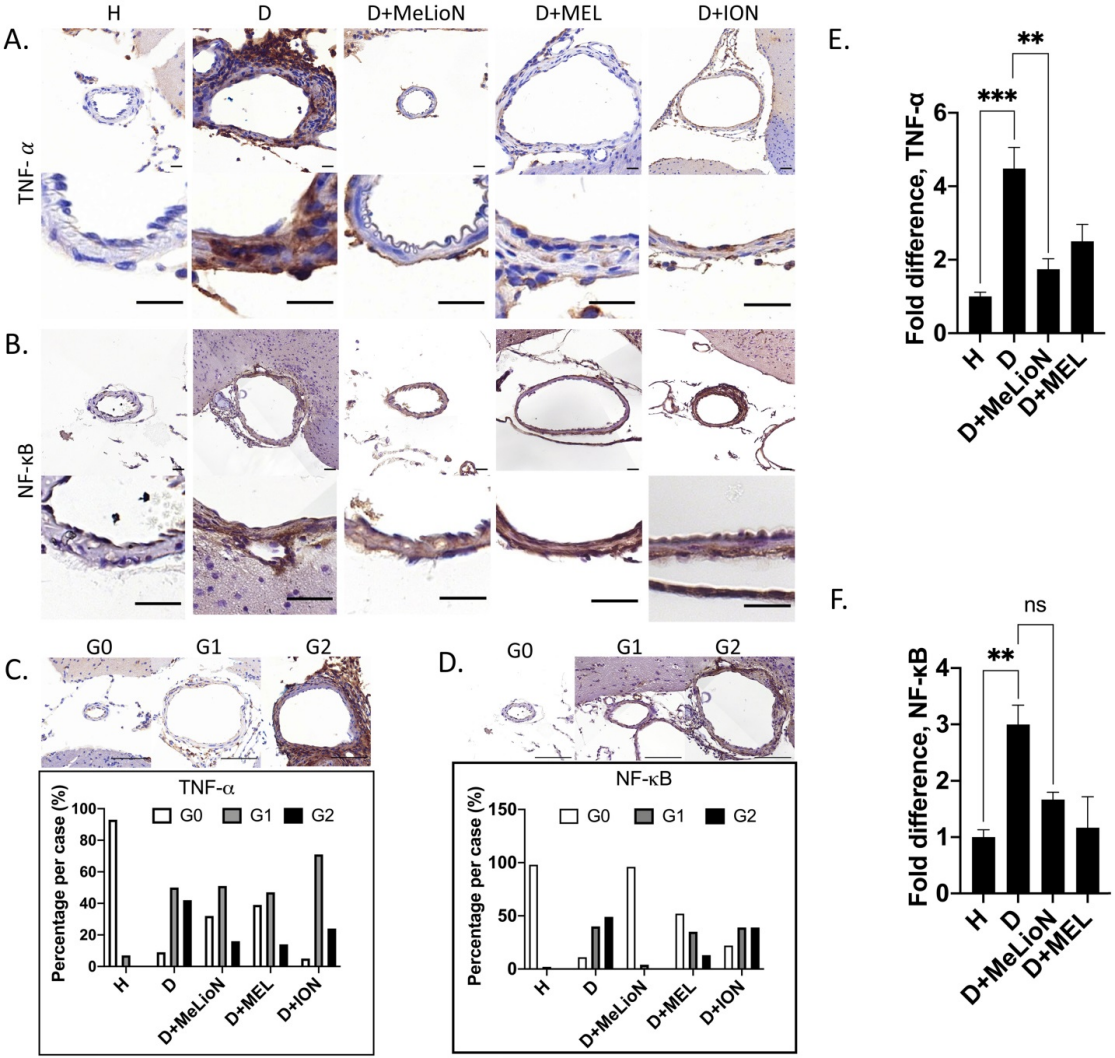
** Melittin-loaded L-arginine-coated iron oxide nanoparticle (MeLioN) blocking the expression of TNF-α and NF-κB in the experimentally induced mouse cerebral arterial wall. (A, B)** Immunohistochemistry staining and **(C, D)** grading of TNF-α and NF-κB expression. MeLioN treatment ameliorated TNF-α and NF-κB expression in the arterial wall. H, healthy control; D, disease control, ION, iron oxide nanoparticle-treated group; MEL, free melittin-treated group; MeLioN, melittin-loaded L-arginine-coated iron oxide nanoparticle-treated group. Scale bar: 20 µm in A and B, 50 µm in C and D. In disease control, the percentile of grade 0 and grade 2 in TNF-α and NF-κB expression was significantly lower and higher than that of healthy control (p<0.0001; p<0.0001; n=6). The MeLioN-treated group exhibited decreased expression of TNF-α and NF-κB, with significantly higher grade 0 and lower grade 2 proportion than disease control (p<0.001; p<0.001 and p<0.0001; p<0.0001, respectively). **(E)** Real-time polymerase chain reaction of TNF-α. TNF-α mRNA expression was 4.5 times up-regulated on three weeks after IADE induction compared to healthy control (*p*<0.001). However, the TNF-α mRNA expression of the MeLioN-treated group reduced to be 33.3% of disease control (*p*<0.01). **(F)** Real-time polymerase chain reaction of NF-κB. NF-κB mRNA expression was three times up-regulated after IADE induction compared to healthy control (*p*<0.01). However, the NF-κB mRNA expression of the MeLioN-treated group reduced to be 50% of disease control (ns).

After three weeks of IADE induction, the effects of MeLioN on the TNF-α and NF-κB signaling pathways in mouse cerebral arterial walls were evaluated by IHC grade and PCR (Figure 7). IHC analysis revealed that MeLioN treatment attenuated TNF-α and NF-κB activation in arterial walls. In term of TNF-α expression in the arterial wall, the grade 0 and grade 2 proportions of disease control were significantly lower and higher than healthy control (8.7% versus 92.6%, *p*<0.0001; 41.7% versus 0.0%, *p*<0.0001; χ^2^ test), respectively. MeLioN-treated group exhibited decreased expression of TNF-α, showing significantly higher grade 0 and lower grade 2 proportion than disease control (32.4% versus 8.7%, *p*<0.001; 16.2% versus 41.7%, *p*<0.001), respectively. The MEL-treated group did not show any difference in TNF-α grade 0 proportion (ns) and grade 2 proportion (ns) as compared to the MeLioN-treated group, respectively. ION group showed a significantly lower proportion of TNF-α grade 0 (*p*<0.001) but no statistical difference in TNF-α grade 2 proportion as compared to MeLioN group (ns), respectively. In term of NF-κB expression in the arterial wall, the grade 0 and grade 2 proportions of disease control were significantly lower and higher than healthy control (10.9% versus 98.0%, *p*<0.0001; 49.1% versus 0.0%, *p*<0.0001; χ^2^ test), respectively. MeLioN-treated group exhibited decreased expression of NF-κB, showing significantly higher grade 0 and lower grade 2 proportion than disease control (96.0% versus 10.9%, *p*<0.0001; 0.0% versus 49.1%, *p*<0.001), respectively. The MEL-treated group showed significant differences in NF-κB grade 0 proportion (52.2% versus 96.0%, *p*<0.0001) and grade 2 proportion (13.0% versus 0.0%, *p*<0.01) as compared to the MeLioN-treated group, respectively. The ION group showed a significantly lower proportion of NF-κB grade 0 (*p*<0.0001) and higher proportion of NF-κB grade 2 as compared to MeLioN group (*p*<0.0001), respectively. In quantitative PCR analysis, TNF-α mRNA expression was 4.5 times up-regulated in three weeks after IADE induction compared to healthy control (*p*<0.001). However, the TNF-α mRNA expression of MeLioN-treated group reduced to be 37.7% of disease control (*p*<0.01). The TNF-α mRNA expression of MeLioN-treated group was not significantly different from that of MEL-treated groups (ns). NF-κB mRNA expression was three times up-regulated after IADE induction compared to healthy control (*p*<0.01). The NF-κB mRNA expression of MeLioN-treated group reduced to be 56.7% of disease control (ns). The NF-κB mRNA expression of MeLioN-treated group was not significantly different from that of MEL-treated group (ns). In Western blot, TNF-α and NF-κB after IADE induction showed thicker band as compared to healthy control, respectively. In both MeLioN and MEL-treated group, the band of TNF-α and NF-κB became as thin as healthy control.

### Co-localization of CD68-positive Macrophage and MeLioN

In the mouse brain after experimental IADE induction and tail vein MeLioN administration, CD68-positive macrophages infiltrated at right Sylvian fissure (Supplementary Figure 10). Prussian blue stain and T2* gradient echo-weighted MRI showed successful MeLioN delivery to the site of arterial inflammation. The CD68-positive macrophage infiltration was co-localized with the MeLioN accumulation at the right Sylvian fissure.

### Cell Viability of MeLioN and free melittin

**Figure 8:**
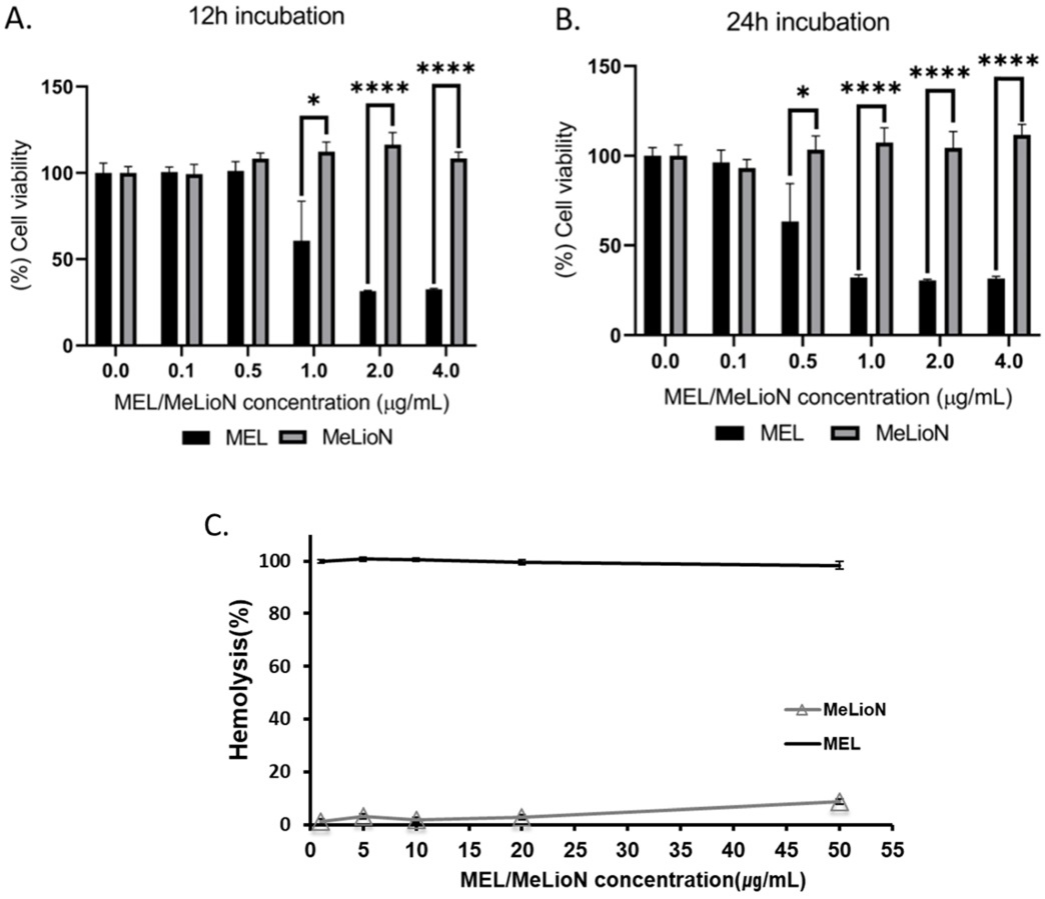
** Cytotoxicity of melittin-loaded L-arginine-coated iron oxide nanoparticle (MeLioN) and free melittin (MEL) using MTT assay and Hemolysis test.** The RAW 264.7 cells were treated with 0.1, 0.5, 1, 2, 4 µg/ml of MeLioN and free melittin for 12 h **(A)** or 24 h **(B)**. In the 12 h of free melittin treatment, RAW 264.7 cell viability was significantly decreased at 1.0 µg/mL, 2.0 µg/mL and 4.0 µg/mL melittin concentrations. In the 24 h of free melittin treatment, the cell viability was significantly decreased at 0.5 µg/mL, 1.0 µg/mL, 2.0 µg/mL and 4.0 µg/mL melittin concentrations. No significant viability changes were detected at melittin concentration below 1.0 µg/mL (for 12 h) and 0.5 µg/mL (for 24 h). In contrast, the 12 h and 24 h of MeLioN treatment did not decrease RAW 264.7 cell viability until full dose escalation to 4 µg/mL of melittin concentration. The cell viability (%) was expressed as mean ± standard error of the mean (n=6) and compared the free melittin and MeLioN-treated groups. ****, *p*<0.001; ***, *p*<0.001; **, *p*<0.01 and *, *p*<0.05. **(C)** The mouse blood was treated with 1, 5, 10, 20, 50 µg/mL of MeLioN and free melittin. Hemolysis (%) was expressed as mean ± standard error of the mean (n=3) and compared the free melittin and MeLioN-treated groups. MeLioN showed no hemolytic activity at up to 20 µg/mL (1-2%) and approximately 10% at 50 µg/mL, while free melittin showed 100% hemolytic activity at from 1 µg/mL.

MTT assay was conducted to determine effects of MeLioN and free melittin on the cell viability at different doses (Figure 8A,B). The RAW 264.7 cells were treated with 0.1, 0.5, 1, 2, 4 μg/mL of MeLioN and free melittin for 12 h or 24 h. In the 12 h of free melittin treatment, RAW 264.7 cell viability was significantly decreased at 1.0 μg/mL, 2.0 μg/mL and 4.0 μg/mL melittin concentrations. In the 24 h of free melittin treatment, the cell viability was significantly decreased at 0.5 μg/mL, 1.0 μg/mL, 2.0 μg/mL and 4.0 μg/mL melittin concentrations. No significant viability changes were detected at melittin concentration below 1.0 μg/mL (for 12 h) and 0.5 μg/mL (for 24 h). In contrast, the 12 h and 24 h of MeLioN treatment did not decrease RAW 264.7 cell viability until full dose escalation to 4 μg/mL of melittin concentration. For hemolytic assay (Figure 8C), mouse blood was treated with 1, 5, 10, 20, 50 μg/mL of MeLioN and free melittin. Hemolysis (%) was expressed as mean ± standard error of the mean (n=3) and compared the free melittin and MeLioN-treated groups. MeLioN showed no hemolytic activity at up to 20 μg/mL (1-2%) and approximately 10% at 50 μg/mL, while free melittin showed 100% hemolytic activity at from 1 μg/mL. *In vitro* drug release experiment showed that approximately 6% to 9% of melittin was released from the MeLioN in pH 7.4 PBS at 37 °C and 4 °C over 2, 4, 8, 24, and 48 h, respectively (Supplementary Figure 8). The culture of RAW 264.7 cells on 48 hours after MeLioN treatment showed that RAW 264.7 cells engulf MeLioN within the cytoplasm (Supplementary Figure 9).

### Concomitant Saccular Cerebral Aneurysm Formation

Concomitant saccular cerebral aneurysms were found in the disease control (9/85; the number of saccular aneurysms out of the observed arterial walls; Supplementary Figure 11) and free melittin-treated group (10/53). However, no saccular aneurysm was found in healthy control (0/71), ION- (0/12), and MeLioN-treated group (0/26).

## Discussion

This proof-of-concept study demonstrated that ION-based melittin delivery prevents dolichoectasia formation in the driving cascades of vascular inflammation and ECM degeneration. MeLioN treatment ameliorated morphologic changes and restored ECM alterations in the cerebral arterial wall of experimentally induced mouse IADE. The arterial diameter and wall thickness of the MeLioN-treated group was significantly smaller and larger than that of disease control, respectively. MeLioN inhibited IADE development more effectively than free melittin in a mouse model. By loading melittin to ION, we were able to reduce MMP activity, inflammatory cytokines, and chemoattractant signals of macrophages with a favorable toxicity profile. To the best of our knowledge, this is the first study to demonstrate the protective effects of melittin on IADE development and to select ION as a nanocarrier platform for the macrophage-targeted melittin delivery.

The arterial walls are composed of SMC, elastin, and collagen to maintain integrity, elasticity, and mechanical strength. The matrix metalloproteinase such as MMP-9 released by activated macrophage digests elastin, leading to SMC apoptosis, and impair the mechanical strength and subsequent dolichoectasia formation 30-32,51. In this experimentally induced mouse model, elastase injection and hypertension induction lead to pathological breakdown of IEL and SMC layer, and remodeling by deposition of collagen fibers. The difference of mouse model from rabbit model may be the technique of basal cistern elastase injection, in that only surgical cut-downs and ligations of the rabbit common carotid arteries lead to acute blood flow augmentation, cerebral artery dilatation, and vascular wall remodeling 48-50. The IEL fragmentation and thinning of medial SMC layer are common in patients with dolichoectasia 33-35, which are consistent features of mouse IADE 30-32.

In this mouse IADE model, endothelial cell dysfunction was induced by the combination of stereotaxic elastase injection into CSF space and hemodynamic stress from continuous angiotensin II infusion, fed with a high salt diet, and unilateral nephrectomy. The hemodynamic stress may lead to the endothelial dysfunction, inflammatory cell infiltrations, phenotypic modulation and degeneration of SMC, remodeling of ECM, cell death and vessel wall degeneration. MCP-1 from pro-inflammatory endothelial cells attracts circulatory monocytes that migrate and adhere to the arterial walls with inflammatory processes.

MMP is the family of proteolytic enzymes that degrade type IV collagen and elastin and mediate the vascular remodeling 52. The macrophages and inflammatory cells secrete MMP-9 leading to ECM degradation and dolichoectasia 31,51,53-58. The immunohistochemistry in this study showed highly expressed MMP-9 in the arterial wall of mouse IADE, which was consistent with findings of RT-PCR. The MMP-9 expression in murine model was in line with the increased serum level of MMP-9 in patients with dolichoectasia 53,54. The clinical series of vertigo patients revealed that high plasma levels of MMP-9 are associated with vertebrobasilar dolichoectasia 53. MMP-9 are also elevated in arterial walls of other vascular diseases with inflammatory processes 55-61. Clinical studies showed elevated serum levels of MMP-9 in the patients who had AAA 57,58, arterial dissection 55,56, and/or intracranial aneurysm 59-61. The coexistence of IADE either with saccular intracranial aneurysm or AAA is relatively common, which may suggest the wide spectrum of MMP-related disease entity 51-61, although it remains unclear whether the IADE and saccular aneurysm formation share a common pathogenetic mechanism. We found concomitant saccular intracranial aneurysm formation in approximately 10-20% of IADE mouse models similar to Zhu et al 31. In contrast to isolated IADE, diffuse IADE might be the vascular phenotype secondary to a systemic arteriopathy affecting multiple vascular beds, and the aneurysmal dilation of entire vascular segments involves two or more cerebral vasculatures. The patients with brain infarction and IADE were more likely to be older in age, male, and have a risk factor of hypertension or previous history of myocardial infarction compared with those without IADE 6,17. The various etiologies may lead to IADE development 62, including Marfan syndrome, Ehlers-Danlos syndrome Type IV, Fabry disease 63, Pompe disease, neurofibromatosis Type I, tuberous sclerosis, moyamoya diseases, PHACE syndrome 64, autosomal dominant polycystic kidney disease, fibromuscular dysplasia, and acquired immune deficiency syndrome 65,66.

MeLioN treatment markedly attenuated MMP-9 activity in arterial walls, supporting the protective role of MeLioN on IADE development. MMP activity in the cerebral arterial wall of MeLioN-treated group was significantly reduced compared to untreated, ION alone- or free melittin-treated group. This mitigated arterial diameter enlargement and wall thinning in the MeLioN-treated group was consistent with downregulated gene expression related to elastin degradation, compensatory collagen overproduction, and SMC apoptosis.

The monocytes and macrophages are key inflammatory modulators performing diverse actions in the IADE formation 31. By double immunostaining with CD45/MAC387 markers, the infiltrated inflammatory cells were confirmed as macrophages. The macrophages release various proteinase and pro-inflammatory cytokines through positive feedback and perturbation of several complex signaling pathways. There has been no report regarding the activation of the cytokines in the arterial walls of dolichoectatic segment, but in this study of dolichoectasia, TNF-α and NF-κB were highly activated. As key regulators, TNF-α and NF-κB play crucial roles either independently or in combination in the initiation of IADE development by inducing macrophage recruitment and activation. This finding showed similarity to intracranial aneurysm wall that activated macrophages secrete TNF-α, which amplify a pro-inflammatory downstream gene expression such as MCP-1, COX2, and MMP-9 either via TNF-TNFR1 67-70 or NF-κB pathways 71-73. MeLioN interrupted the feedback loop between inflammation and macrophages, as evidenced by the decreased TNF-α and NF-κB, further corroborated by the decreased MMP-9 activity. MeLioN-treated mice reduced the number of macrophages, levels of MCP-1 and MMP-9, achieving protection from dolichoectasia formation. This protective role of melittin in the IADE formation was compatible with the previous studies reporting melittin's anti-inflammatory actions. Melittin exerted mitigative effects for several inflammatory diseases via abrogating myriad feedback loops between cytokine and macrophage 36-40. Melittin is the primary ingredient of honeybee venom (Apis mellifera), consisting of 26 amino acid residues (NH2-GIGAVLKVLTTGLPALISWIKRKRQQ-CONH2) 36-42. Transdermal injection of bee venom by acupuncture or direct bee stings has been performed for apitherapy. Intravenous administration of high dose melittin for clinical application was hampered due to the serious adverse reactions, including pain, hemolysis, and anaphylaxis 26-30. The cytolytic peptide melittin physically disrupts the cell membrane rather than interacting with a specific binding site. Melittin was loaded on the surface of ION as a core-shell structure to circumvent its side effect, which widened the safe dose range of intravenous administration. While several types of melittin nanoparticles have been introduced so far 41-45, ION was selected as the nanocarrier platform with the advantage of feasible clinical application of drug delivery and MRI 74,75. Here, we demonstrated that melittin loaded within ION decreased pro-inflammatory cytokines and suppressed macrophage proliferation in cerebral arterial walls with a favorable toxicity profile. The blood counts were within the normal range. There was no sign of severe organ toxicity in the blood enzyme tests suggesting liver and kidney damage except transient BUN elevation. The Hematoxylin and Eosin stain of major organs showed no sign of inflammation, necrosis, or fibrosis. On the hemolysis test, MeLioN does not cause hemolysis, while free melittin results in hemolysis. According to the MTT assay, no cytotoxicity was found in MeLioN-treated cells even in the higher dosage that marked cytotoxicity was observed in free melittin-treated cells. MeLioN is inferred not to perforate cell membrane differently from the free melittin peptide actions. Instead, MeLioN might act as a nanoparticle unit with characteristics different from mere conjugation of melittin and iron oxide nanoparticle. The free melittin release from MeLioN was measured less than 10% in 48 hours. Without cell membrane perforation, the MeLioN can be delivered into target lesion by various nanoparticle-engulfing mechanisms of monocyte or macrophages as seen from the confocal microscopy of macrophage cultures with MeLioN, which requires further investigation in the future study. MeLioN showed more efficacy in the anti-inflammatory action than free melittin, and reduced macrophage infiltration and related pro-inflammatory cytokines release, while ION alone without loading of melittin did not show any therapeutic effect.

On histology and MRI, CD68-positive macrophage infiltrations were co-localized with the MeLioN distributions on the Sylvian cistern of the mouse brain, suggesting the potential role of MeLioN-harboring macrophage. We showed that the brain tissue near to the elastase-injected area was stained positive for both CD68 and Prussian blue after ION administration. This was supported by ICP-MS measurement of brain iron concentration showing temporal peak on one day and clearance over a month after MeLioN administration. After intravenous administration, MeLioN can be taken up by various types of cells including circulating monocytes, tissue macrophage, other neutrophils, SMCs, and endothelial cells. It is not clear whether MeLioN is taken mainly by pre-existing tissue macrophage, or whether circulating monocytes on the way of recruitment into inflamed arterial wall deliver MeLioN after engulfment of MeLioN in circulation, which needs to be further elucidated.

The authors acknowledge that there are several limitations to be further studied. First, physicochemical characteristics of MeLioN including particle size, surface charge and hydrophobicity might have potentially affected the biosafety and efficacy. In this study, L-arginine was used as a coating agent of the bare Fe_3_O_4_ nanoparticle to prevent the aggregations due to the strong magnetic dipole-dipole attractions between particles, which stabilize the nanosuspension as well as create binding sites for melittin. In the measurement of the hydrodynamic size of MeLioN over the fourteen days varying the solvents, the MeLioN dispersed in the deionized water showed stable hydrodynamic size, while the MeLioN either dispersed in the PBS or normal saline showed aggregations. Based on this stability data, the MeLioN dispersed in the deionized water was injected via the mouse tail vein, which showed no sudden death or tail necrosis related to the total of five sessions of MeLioN administration. MeLioN has a positively charged surface charge but did not cause serious side reactions after tail vein injection might be due to “protein corona” formation by the adsorption of serum proteins to the positively charged MeLioN surface and preventing inter-particle interaction 76, 77. However, further investigations are necessary to confirm the *in vivo* surface charge and hydrodynamic size of MeLioN after intravenous injection. Second, the blood-brain barrier (BBB) penetration of MeLioN was not assessed, although it might be an important issue. We admitted that the BBB permeability might be increased in the dolichoectasia model created by cisternal elastase injection, and it may affect the enhanced delivery of MeLioN in this study. The increased BBB permeability in the dolichoectasia model may make it difficult to assess the true ability of MeLioN to cross the BBB. However, because we hypothesize that the dolichoectasia developments are mediated by the recruitment of monocytes and macrophages that occur without crossing the BBB, the BBB penetration of MeLioN may be beyond the scope of this study. The BBB penetration of MeLioN needs to be further investigated in the next study. Third, we injected elastase into the right basal cistern, but Dai et al. 30-32 delivered elastase through cisterna magna considering the more frequent occurrence of IADE in the posterior circulation and the basilar artery. We need to further compare the potentially distinctive IADE model features depending on the various elastase administration methods. Fourth, the experimental mouse model resembles key histological features that are observed in the human IADE, but the outcome variability of elastase-induced IADE development in mice may depend on the genetic background and show sexual dimorphism 32. The differences in sex and strain need to be further considered in the outcome assessment. Fifth, the variability in measurement of diameter and thickness as per the anatomic location was not considered due to the small number of specimens, but the average differences among study groups reached the sufficient range of statistical significance. Sixth, the decreased the infiltrations of CD68-positive macrophage suggested the suppressed proliferation of macrophages in MeLioN-treated group, but the phenotypic change of macrophage by MeLioN, such as M1/M2 polarity shift 78,79, was not evaluated in this study. Further investigations are needed to clarify the phenotypic change of macrophage by MeLioN.

Taken together, the present study of the mouse IADE model demonstrated that macrophage infiltration into the arterial wall and up-regulation of pro-inflammatory mediators including MCP-1, TNF-α, and NF-κB. They also played critical roles on the MMP-9 secretion and ECM remodeling related to the IADE formation. MeLioN designed for ION-mediated melittin delivery effectively prevents the IADE formation by suppressing macrophage-mediated inflammatory responses and subsequently attenuating MMP activity and ECM remodeling. Given the enhanced safety profile and delivery rate of this nanocarrier platform, MeLioN can be a promising preemptive strategy protecting IADE development. A further clinical feasibility study would be necessary for the high-risk population of developing IADE.

## Supplementary Material

Supplementary figures and table.

## Abbreviations

AAA: abdominal aortic aneurysm
ANOVA: analysis of variance
BCA assay: bicinchoninic acid assay
CD68: cluster of differentiation 68
ECM: extracellular matrix
FESEM: field emission scanning electron microscopy
FETEM: field emission transmission electron microscopy
FT-IR: fourier-transform infrared spectroscopy
FT-UV-VIS-IR: fourier-transform ultra-violet visible infrared spectroscopy
GAPDH: glyceraldehyde 3-phosphate dehydrogenase
IADE: intracranial arterial dolichoectasia
IHC: immunohistochemistry
ION: iron oxide nanoparticle
LION: L-arginine-coated iron oxide nanoparticle
MCP-1: monocyte chemoattractant protein 1
MEL: melittin
MeLioN: melittin-loaded L-arginine-coated iron oxide nanoparticle
MMP-9: matrix metallopeptidase 9
MRI: magnetic resonance imaging
MTT assay: 3-(4,5-dimethylthiazol-2-yl)-2,5-diphenyl-2H-tetrazolium bromide
NF-κB: nuclear factor kappa-light-chain-enhancer of activated B cells)
ROI: region of interest
RT-PCR: real time polymerase chain reaction
SMC: smooth muscle cells
TBS: tris-buffered saline
TNF-α: tumor necrosis factor alpha
XRD: x-ray diffraction
α-SMA: alpha smooth muscle actin
