# Supplementary material for: Melittin-loaded Iron Oxide Nanoparticles Prevent Intracranial Arterial Dolichoectasia Development through Inhibition of Macrophage-mediated Inflammation
Source: Int J Biol Sci. 2021 Sep 3;17(14):3818–36. doi: 10.7150/ijbs.60588 (PMC8495379; doi:10.7150/ijbs.60588)
Supplement: Supplementary file 1 — Supplementary figures and table. [file ijbsv17p3818s1.pdf]

Supplementary table and figures

Supplementary table 1. Primer sequences for real time polymerase chain reaction of mouse brain. TNF-α, tumor necrosis factor-α; NF-κB, nuclear factor kappa-light-chain-enhancer of activated B cells; MCP-1, monocyte chemoattractant protein-1; MMP-9, matrix metalloproteinase-9; GAPDH, Glyceraldehyde 3-phosphate dehydrogenase.

**Genes**	**Primer sequence (5' → 3')**	**Annealing temperature (℃)**
TNF-α	F: CATCCGTTCTCTACCCAGCC	59
	R: AATTCTGAGCCCGGAGTTGG	
NF-κB	F: CCTTGAAGGGATTTCCCTCC	58
	R: GGAGGGAAATCCCTTCAAGG	
MCP-1	F: TGATCCCAATGAGTAGGCTGGAG	60
	R: ATGTCTGGACCCATTCCTTCTTG	
MMP-9	F: GCCACTACTGTGCCTTTGAGTC	60
	R: CCCTCAGAGAATCGCCAGTACT	
GAPDH	F: AGTGCCAGCCTCGTCTCATA	58
	R: GGTAACCAGGCGTCCGATAC	

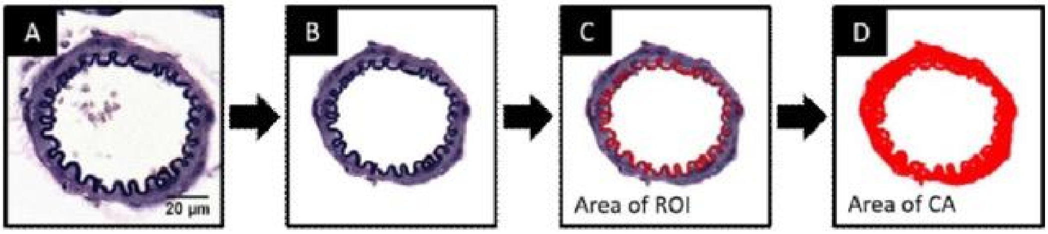

Supplementary figure 1. Quantification method of arterial wall component. After trimming background (A, B), the elastin component with blue pixel on Elastica van Gieson stain was segmented to the area of a region of interest (C; ROI in red) by using thresholding function in ImageJ (NIH, Bethesda, MD, USA; https://imagej.nih.gov/nih-image/). The total cerebral arterial wall was segmented to the area of CA (D; in red). The percentage of elastin in the arterial wall was calculated by $area_{ROI}/area_{CA}$ x 100%.

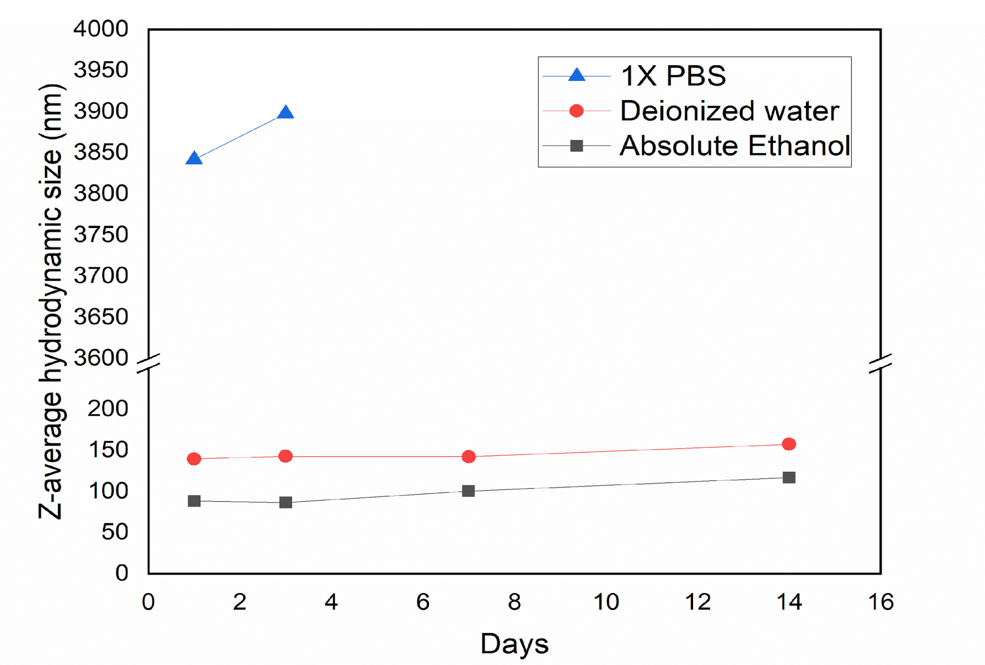

Supplementary figure 2. Hydrodynamic size change of MeLioN in four solvents over fourteen days. A 0.1 mL volume of MeLioN (1.25 mg/mL of L-arginine coated iron oxide nanoparticles with 0.3 mg/mL of melittin loading) was dispersed in 2.5 mL of each solvent such as deionized water, absolute ethanol, 1X PBS and 0.9% sodium chloride, respectively. A 2.5 mL is the average blood volume of mice. 1X PBS solution is composed of 137 mM NaCl, 2.7 mM KCl, 10 mM Na_2HPO_4 and 1.8 mM KH_2PO_4 (pH = 7.4). The nanosuspension in each solvent was measured at one day, three days, seven days, and 14 days after dispersing into the solvent. The hydrodynamic size of MeLioN in 1X PBS and 0.9% sodium chloride at day seven and day 14 were not recorded due to the whole nanoparticles aggregating to the bottom of the tube. The hydrodynamic size of MeLioN dispersed in 0.9% sodium chloride on the first and third day were recorded at 4642 nm and 4398 nm, respectively (Data not shown).

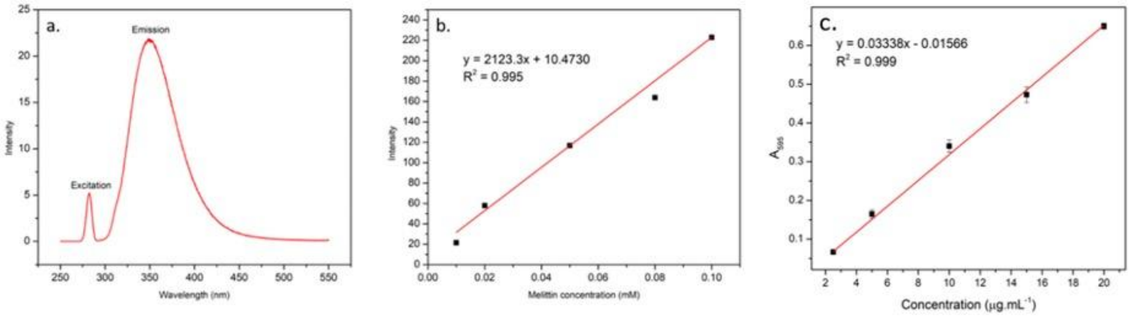

Supplementary figure 3. Melittin concentration of melittin-loaded L-arginine-coated iron oxide nanoparticles (MeLioN). Using tryptophan-fluorescence (A) and Bradford method (B), the amount of melittin within MeLioN was measured as 246.52 ± 0.59 μg/mL and 256.79 ± 1.95 μg/mL, respectively. From the fluorescence intensity curves of five melittin concentration points with tryptophan-sensitive excitation (280 nm) and emission (350 nm) peaks, the calibration curve was derived relating melittin concentration to the fluorescence intensity, and the melittin concentration was determined based on tryptophan-sensitive fluorescence signal intensity of MeLioN. In the Bradford method, the concentration of loaded melittin in MeLioN was measured by mixing the known amount of melittin (0.5 mL, 0.36 mM) and LION (0.5 mL, 2.5 mg/mL) into the centrifuge tube. Centrifuge separated the supernatant and the pellet, and the unbound melittin in the supernatant was analyzed by reacting with Coomassie Brilliant Blue G-250 at room temperature for 5 min. The concentration of melittin is calibrated from the absorbance of the wavelength at 595 nm. The amount of melittin loaded on ION was calculated by subtracting the free melittin unbound to LION ($C_{free\ MEL/supernatant}$) from the given known amount of melittin ($C_{MEL/initially\ added}$) by using equation:

$$C_{MEL/MeLioN} = C_{MEL/initially\ added} - C_{free\ MEL/supernatant}.$$

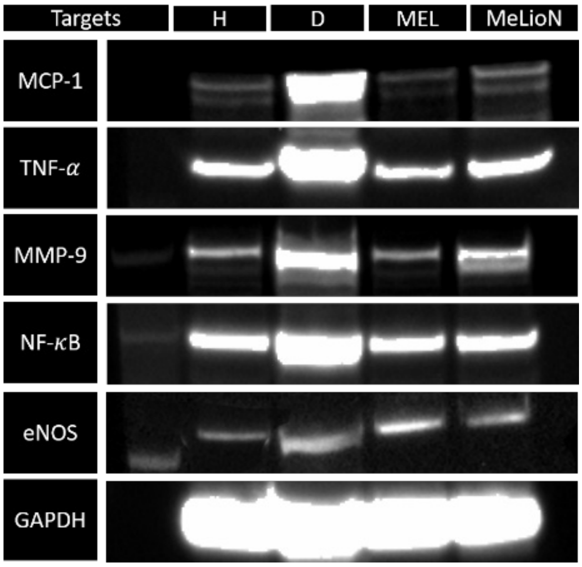

Supplementary figure 4. Western blot of MCP-1, TNF-α, MMP-9, NF-κB, and eNOS three weeks after experimental dolichoectasia induction. The bands of disease control were more intense than healthy control. The bands were indistinct in both MeLioN- and MEL-treated groups.

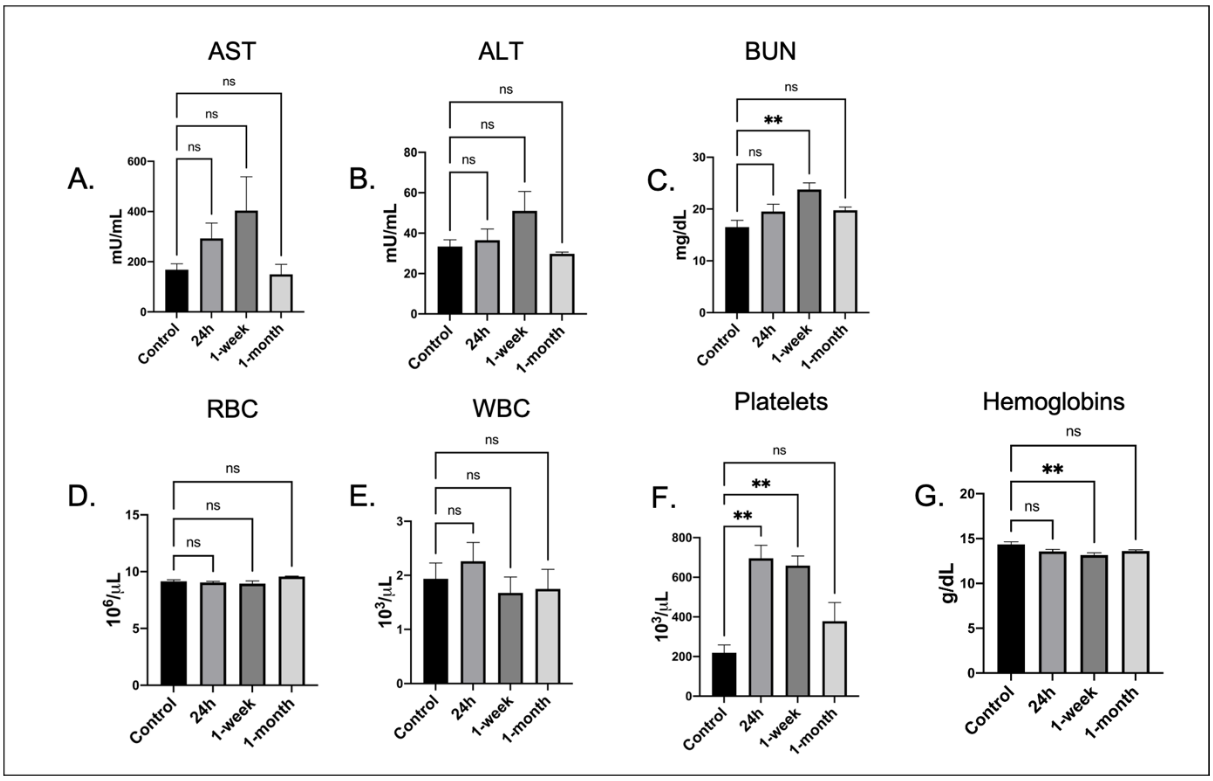

Supplementary figure 5. Biosafety profiles of MeLioN. Blood samples were evaluated in healthy mice in 24 hour, 1-week, and 1-month after a single dose injection of 0.1 mL MeLioN (0.3 mg/mL melittin, 1.25 mg/mL LION). Figure 5A – 5C showed the results of AST, ALT and BUN levels in each study group. Figure 5D – 5G presented the complete blood counts in each study group. The data was presented as mean±SEM (n=4). One-way ANOVA was used and followed by a Dunnett's post-hoc comparison to calculate the *p*-value (*, $p<0.05$; **, $p<0.01$; ***, $p<0.001$; ****, $p<0.0001$; ns, not significant). LION: L-arginine-coated iron oxide nanoparticles. BUN: blood urea nitrogen.

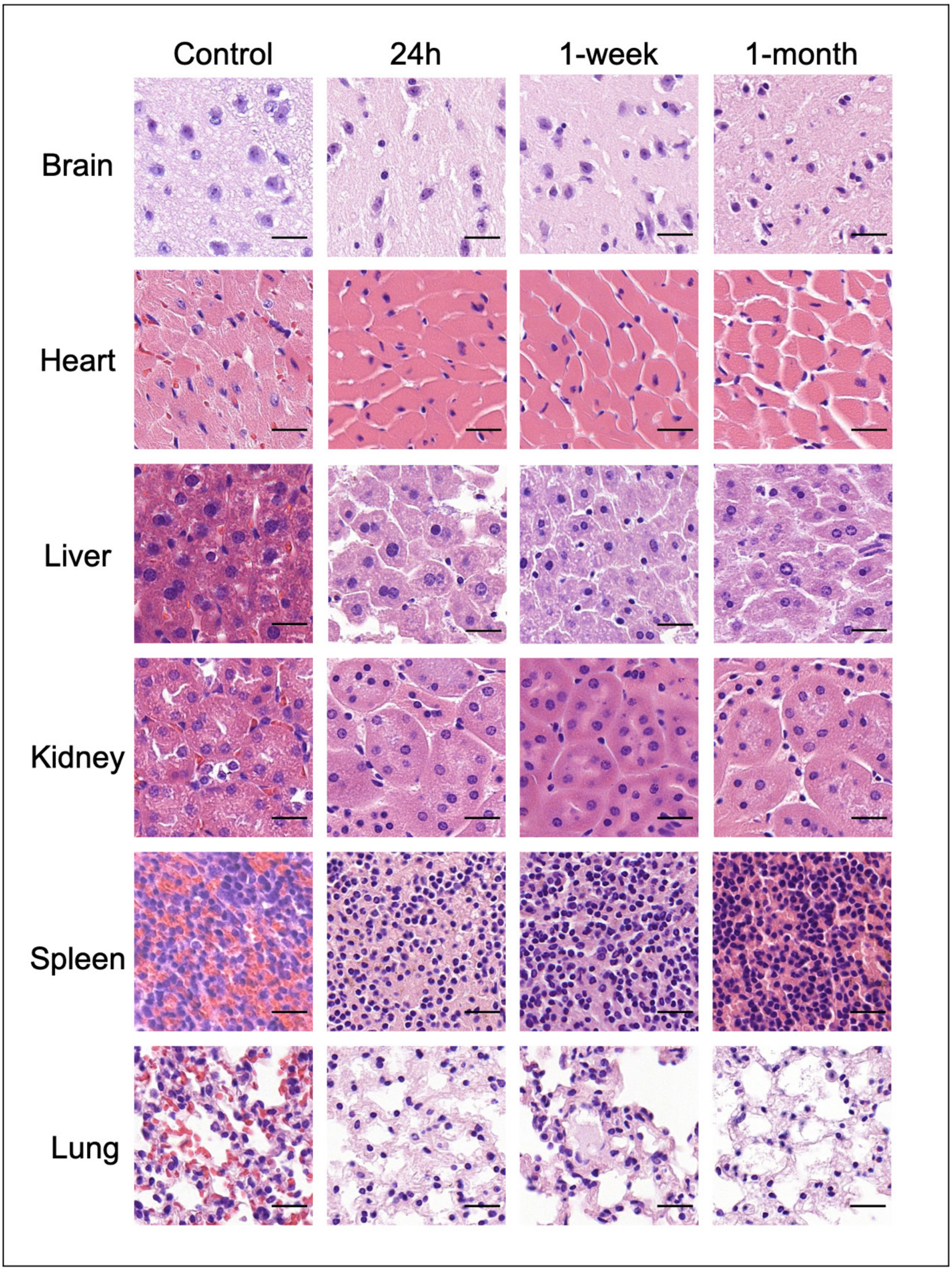

Supplementary figure 6. Hematoxylin and Eosin stain showing no evidence of inflammatory cell infiltrations, necrosis, or fibrotic changes. The major organs, including brain, heart, liver, kidney, spleen, and lung, were collected at the 24-hour, 1-week, and 1-month after MeLioN administration. Scale bar = 20 μm.

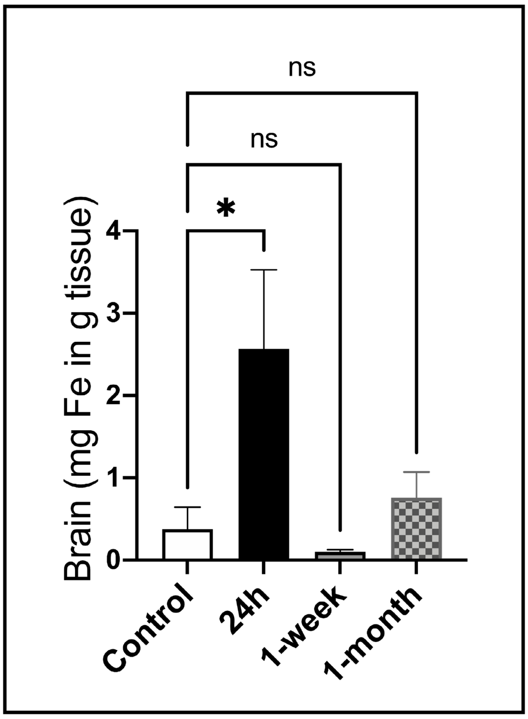

Supplementary figure 7. Brain clearance of MeLioN by measuring total iron content in the brain on one day, 1-week, and 1-month after a single intravenous injection of 0.1 mL MeLioN (0.3 mg/mL melittin, 1.25 mg/mL LION). The data were expressed as mean ± SEM. A one-way ANOVA test was used and followed by a Dunnett's post-hoc comparison to calculate the p-value (*, p<0.05; ns, not significant).

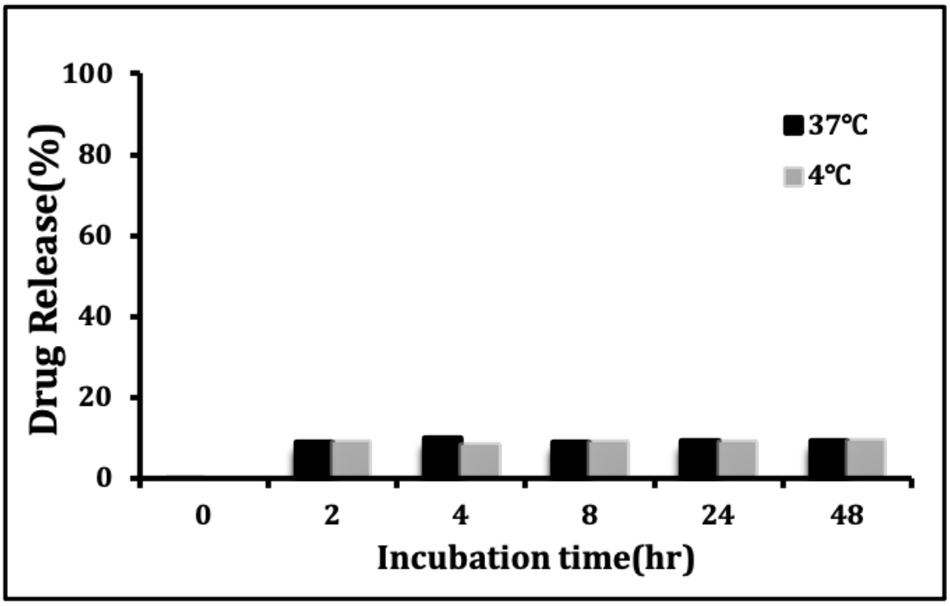

Supplementary figure 8. In vitro melittin release from MeLioN during 48 hours in pH 7.4 PBS at 37 °C and 4 °C.

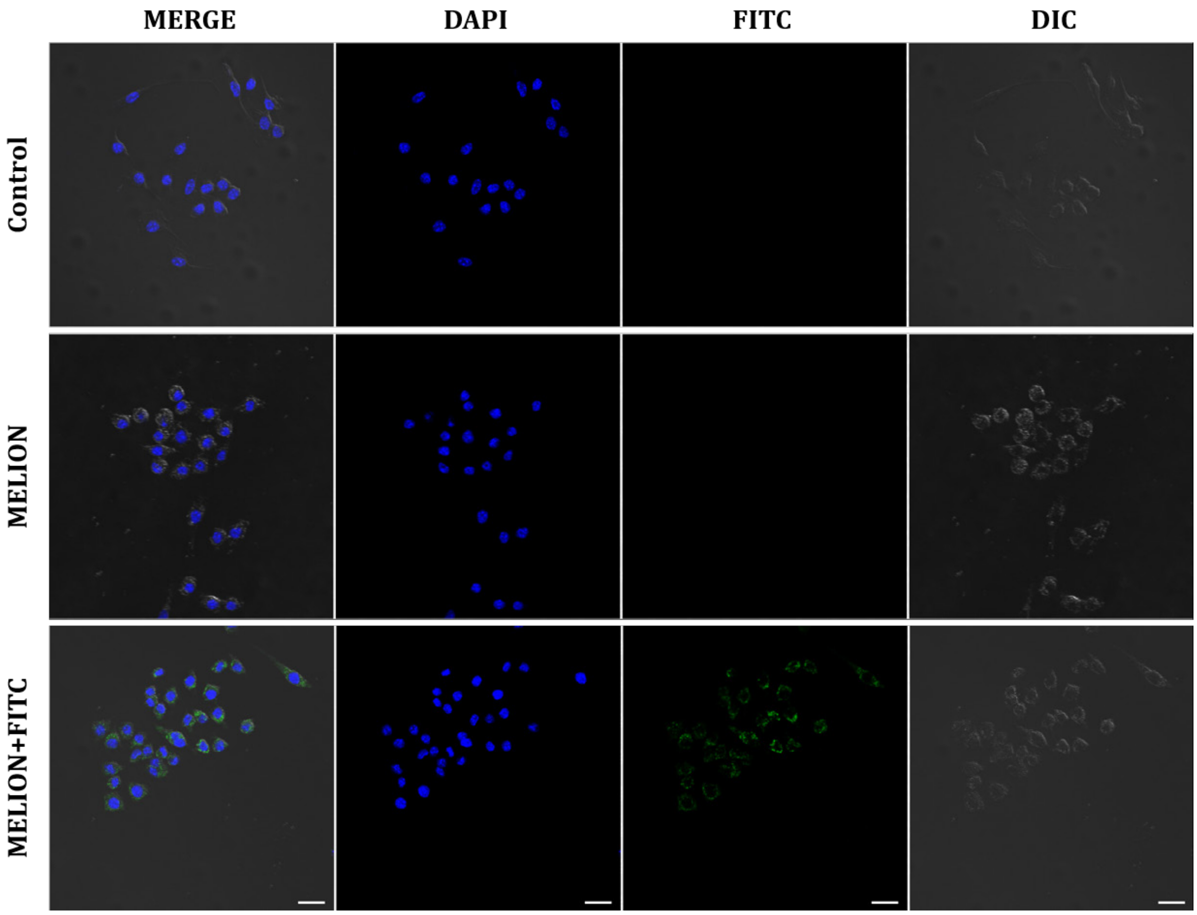

Supplementary figure 9. Immunofluorescence microscopic images of the RAW 264.7 cells engulfing MeLioN in 48 hours. MeLioN- and FITC-conjugated MeLioN-treated group was compared to the control group, respectively. Merged images combined three color channels. DAPI (4′,6-diamidino-2-phenylindole) was used to stain nuclei (blue) of the cell. FITC (fluorescein isothiocyanate) represents the existence of MeLioN in the sample (green). DIC (differential interference contrast) was used to enhance the contrast in unstained, transparent samples. Scale bar = 20 μm.

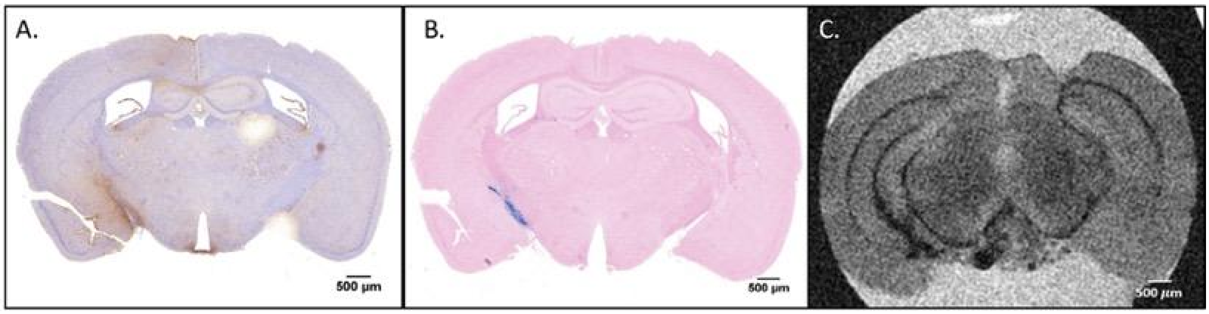

Supplementary figure 10. Co-localization of CD68-positive macrophage and melittin-loaded iron oxide nanoparticle (MeLioN) in the mouse brain after experimental dolichoectasia induction and tail vein MeLioN administration. On CD68 stain (A), CD68-positive macrophages infiltrations were identified at the right Sylvian fissure. On Prussian blue stain (B) and T2* gradient echo-weighted MRI (C), the iron oxide nanoparticle accumulations which is the core component of MeLioN were detected at the right Sylvian fissure. This co-localization between the CD68-positive macrophage infiltration and the MeLioN distribution suggests the potential role of macrophage harboring MeLioN. Scale bar = 500 µm.

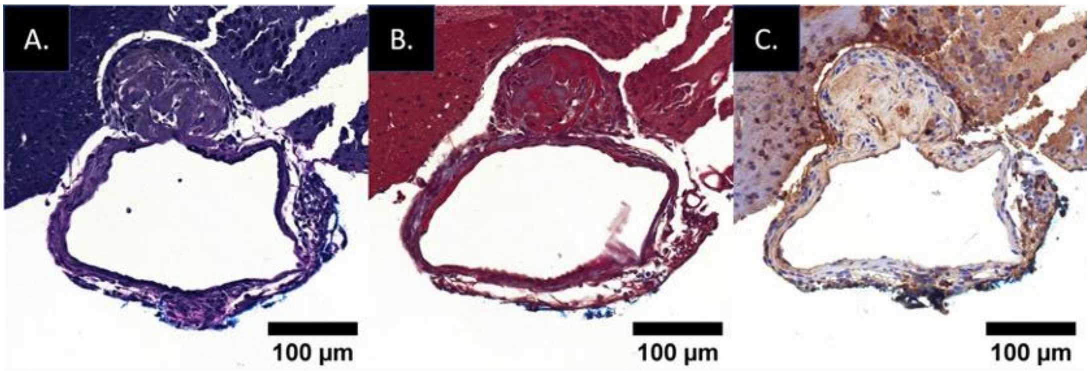

Supplementary figure 11. Saccular mouse cerebral aneurysm with pathologic extracellular matrix remodeling and CD68-positive macrophage infiltration three weeks after experimental dolichoectasia induction. The immunohistochemistry staining of Verhoeff-Van Gieson (A), Trichrome (B), and CD68 (C) showed that saccular arterial wall bulge with internal elastic lamina loss, pathologic collagen production, and CD68-positive macrophage accumulation. Scale bar = 100 µm.
